# A Review of Mechanical Degradation, Interfacial Nonlinear Dynamics and Multi-Bolt Coupling Degradation Mechanisms of Flange-Bolted Joints

**DOI:** 10.3390/s26175533

**Published:** 2026-08-31

**Authors:** Xiaofei Feng, Ming Guo, Shengao Wang, Xiaohan Lu, Yilong Liu, Ziwei Li, Wenjuan Wang, Zijian Xu, Yuqing Liu

**Affiliations:** Naval University of Engineering, Wuhan 430033, China

**Keywords:** bolted flange joint, preload relaxation, rotational self-loosening, microslip, stiffness degradation, Iwan model, parameter identification, multi-bolt interaction, hysteresis constitutive model

## Abstract

Bolted flange joints are critical load-bearing connection components of complex mechanical equipment, whose service performance is dominated by the coupled evolution of bolt preload relaxation and interfacial stiffness degradation under long-term cyclic combined loads. Under cyclic transverse excitation, the contact interfaces experience successive full-stick, partial microslip and macroslip states, accompanied by embedding, creep and friction-induced wear, resulting in time-varying preload attenuation and the continuous degradation of joint mechanical properties. Distinguishing preload relaxation without nut rotation from rotational self-loosening is essential for revealing multi-bolt flange degradation mechanisms. This paper presents a systematic review of physics-driven constitutive modeling and degradation characterization of bolted flange connections. A unified classification framework for joint interface models is established, covering static friction models; velocity-dependent dynamic friction models; hysteresis stick–slip models, represented by the four-parameter Iwan model and Valanis endochronic model; and reduced-order equivalent joint models. The inherent differences between the Dahl model and LuGre model regarding the Stribeck velocity–friction characteristics are clarified. The representative models are comprehensively evaluated from the perspectives of physical interpretability, hysteresis reproduction accuracy, preload–degradation correlation and engineering applicability. A further comparative analysis is conducted of parameter identification bottlenecks of the Iwan, LuGre and Valanis models, focusing on multi-solution risk, anti-noise robustness and cross-working-condition transferability. Compared with single-bolt lap specimens, multi-bolt annular flanges exhibit prominent inter-bolt elastic interaction, circumferential contact pressure non-uniformity and local-to-global chain-reaction degradation behaviors. The common experimental test-beds and sensing techniques for joint degradation monitoring are summarized, and the measurement limitations, including sensor drift, synchronization error and installation constraints, and their influences on model parameter identification, are discussed. The existing machine-learning, digital-twin and physics-informed modeling applications for bolted joints are briefly outlined. Finally, this review summarizes the existing research consensus, unresolved contradictions and open research challenges. Special attention is paid to the limitations of existing time-variant Iwan-type models for multi-bolt flange degradation. Potential future research directions include multi-channel synchronous sensing matching time-varying constitutive models, quantification of inter-bolt load redistribution, and robust identification strategies under noisy experimental data.

## 1. Introduction

As the core area of the manufacturing sector, complex equipment serves as a critical metric for evaluating a nation’s industrial capacity and overall national strength, as well as the cornerstone of national security. China’s complex equipment manufacturing industry has sustained rapid growth in recent decades. It now ranks among the world’s leaders in total industrial output, while achieving remarkable advances in industrial structural upgrading and core technological breakthroughs. Modern complex machinery is evolving toward intelligent autonomy, energy-efficient green manufacturing, unmanned collaborative operation, extreme environmental adaptability, and cross-disciplinary integration. Against this backdrop, growing research and engineering attention is being devoted to the comprehensive performance of complex equipment, with structural safety and long-term reliability serving as the most critical indicators of operational viability.

### 1.1. Systematic Review Methodology

This systematic literature review strictly complies with the PRISMA 2020 statement to achieve transparent, fully reproducible literature retrieval and screening procedures. The complete database retrieval parameters, separate database statistical data, multi-stage screening quantities and standardized exclusion criteria are fully presented below.

#### 1.1.1. Retrieval Databases

**Figure 1:**
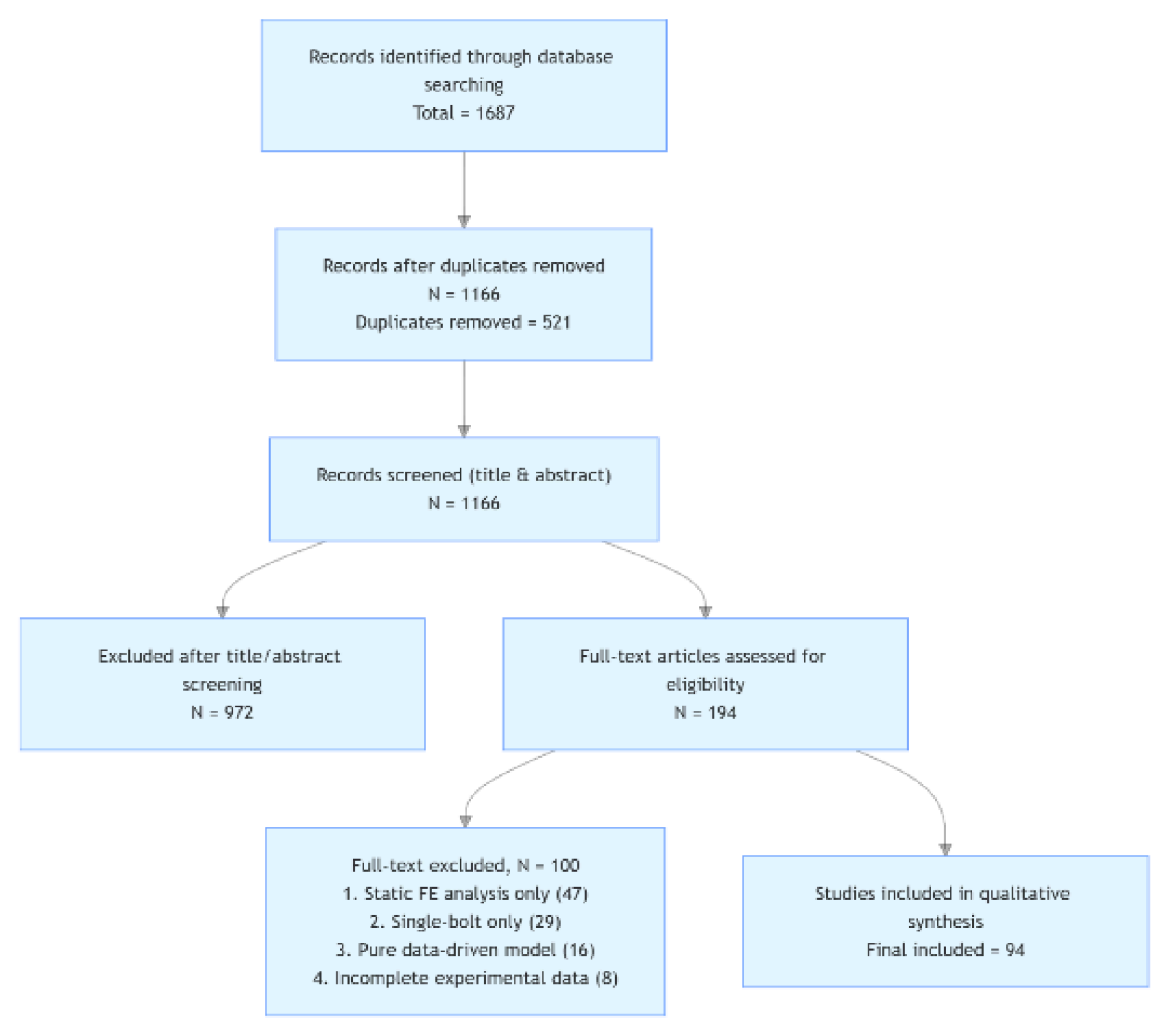
PRISMA 2020 flowchart illustrating the four-stage literature retrieval and screening process, including the quantitative statistics of initial records, duplicate elimination, title–abstract exclusion, full-text elimination with four categorized removal reasons, and the final number of included studies.

Five authoritative global academic databases covering mechanical vibration, tribology, pressure vessel engineering and structural health monitoring were selected for a parallel literature retrieval: Web of Science Core Collection, ScienceDirect, ASME Digital Collection, CNKI (China National Knowledge Infrastructure), and IEEE Xplore. The separate retrieval record statistics for each database are provided in the PRISMA flowchart (Figure 1).

#### 1.1.2. Combined Search Strings

The retrieval keywords were divided into three groups: flange joint degradation, interfacial hysteresis friction model and bolt preload evolution. The unified combined search string applied across all databases is listed below.

English search string:

(flange bolted joint OR annular flange connection) AND (preload relaxation OR bolt self-loosening OR stiffness degradation OR interfacial microslip) AND (Iwan model OR friction hysteresis model OR nonlinear dynamics OR coupling degradation).

Chinese search string (for CNKI only):

法兰连接 OR 螺栓法兰结构 AND 预紧力松弛 OR 螺栓松动 OR 刚度退化 OR 界面微滑移 AND Iwan 模型 OR 摩擦迟滞模型 OR 非线性动力学 OR 多螺栓耦合退化.

#### 1.1.3. Retrieval Time Window and Cut-Off Date

The retrieval time range was set from January 1990 to 10 July 2026; 10 July 2026 was confirmed as the final literature cut-off date, and no newly published documents after this date were included.

#### 1.1.4. Four-Step Quantitative Screening Statistics

The total raw records retrieved from the five databases totaled 1687 papers. The separate record numbers for each database are marked in the attached PRISMA flowchart (Figure 1).

Automatic deduplication was conducted via EndNote 20 reference management software, followed by a manual double verification of title, author and publication year to eliminate repeated documents. A total of 521 duplicate records were removed, leaving 1166 unique non-repetitive papers for preliminary screening.

Two independent reviewers completed a blind screening of the remaining 1166 articles. A total of 972 papers were eliminated based on three unified exclusion criteria:

1. Irrelevant research objects: Single-bolt lap beams without annular flanges, pure non-bolt friction test pieces;

2. Mismatched research theme: Only static strength calculation without cyclic load degradation analysis or independent material fatigue research without contact interface friction evolution;

3. Non-standard document types: Patents, conference abstracts without complete full text, textbooks, and industrial design specifications. After this stage, 194 candidate full-text papers were retained for a secondary full-text evaluation.

All 194 full-text documents were read and judged thoroughly; 100 papers were eliminated based on four classified elimination reasons:

Forty-seven papers only carried out a finite element static stress analysis, without any discussion of cyclic load-induced interfacial degradation evolution laws;

Twenty-nine papers only considered simplified single-bolt specimens, without research on the annular flange ring deformation or multi-bolt coupling interaction;

Sixteen papers merely built data-driven prediction models without a physical interface constitutive mechanism analysis;

Eight papers contained incomplete experimental data and unverified model conclusions with insufficient reliability. Finally, 94 qualified full-text papers were selected as the formal literature basis of this review.

Available for peer verification upon editorial request. A standard PRISMA 2020 four-step quantitative flowchart (Figure 1) is included to visually summarize all the screening numerical data.

**Figure 2:**
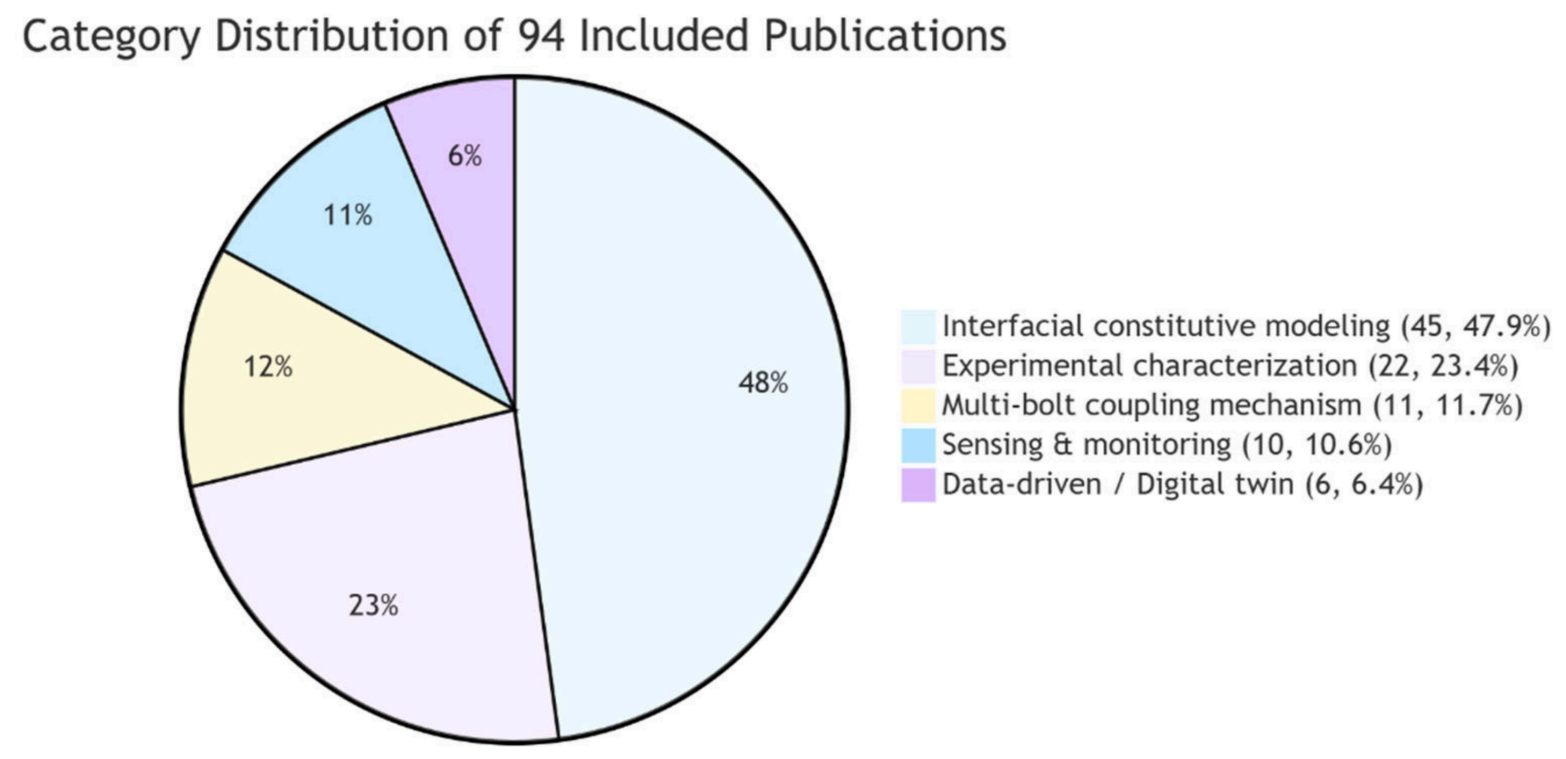
Category distribution pie chart of the 94 included publications, divided into five research branches: friction constitutive modeling, experimental characterization, multi-bolt coupling degradation, sensing monitoring, and data-driven digital twin research. The proportions reflect the current research hotspots and understudied topics in the flange-bolted joint degradation field.

To further improve the transparency of the literature basis supporting each review module, all 94 finally included qualified publications are classified into five research categories according to their core research objectives, and the quantitative distribution and proportional composition are summarized as follows (Figure 2):

Interfacial friction constitutive modeling research: Forty-five papers, accounting for 47.9% of the total included literature. This category covers static friction models; dynamic friction models; hysteresis microslip models, such as the Iwan and Valanis frameworks; and is the dominant research hotspot in existing flange joint degradation studies, providing sufficient theoretical evidence for Section 3.

Experimental characterization of bolted flange degradation: Twenty-two papers, accounting for 23.4%. These documents involve standard benchmark specimens and multi-condition test platforms, which form the experimental data basis for Section 2; however, they lack full-scale multi-bolt flange cyclic test research.

Multi-bolt coupling degradation mechanism research: Eleven papers, accounting for 11.7%. The literature quantity is relatively scarce, indicating that the group-coupled degradation law of annular flanges is still an insufficiently explored research branch, which explains the corresponding research gaps summarized in Section 4.3.

Sensing and structural health-monitoring research: Ten papers, accounting for 10.6%. The existing studies mainly focus on single-bolt preload measurement, while synchronous multi-channel monitoring schemes for full-circle multi-bolt flanges remain limited.

Data-driven, physics-informed model and digital twin research: Six papers, accounting for 6.4%. The relevant research is still in the preliminary stage, with few validations targeting multi-bolt flange engineering components.

From the classification statistics above, it can be seen that the existing literature relies heavily on single-bolt friction constitutive modeling, while studies focusing on multi-bolt coupling, synchronous sensing monitoring and data-driven degradation prediction are relatively scarce. This distribution characteristic matches the core research gaps and future outlooks summarized in Section 5, and verifies that the literature foundation can fully support a discussion of mainstream model theories, while highlighting the insufficient evidence base for multi-bolt and sensing-related research directions.

### 1.2. Engineering Background and Research Significance

**Figure 3:**
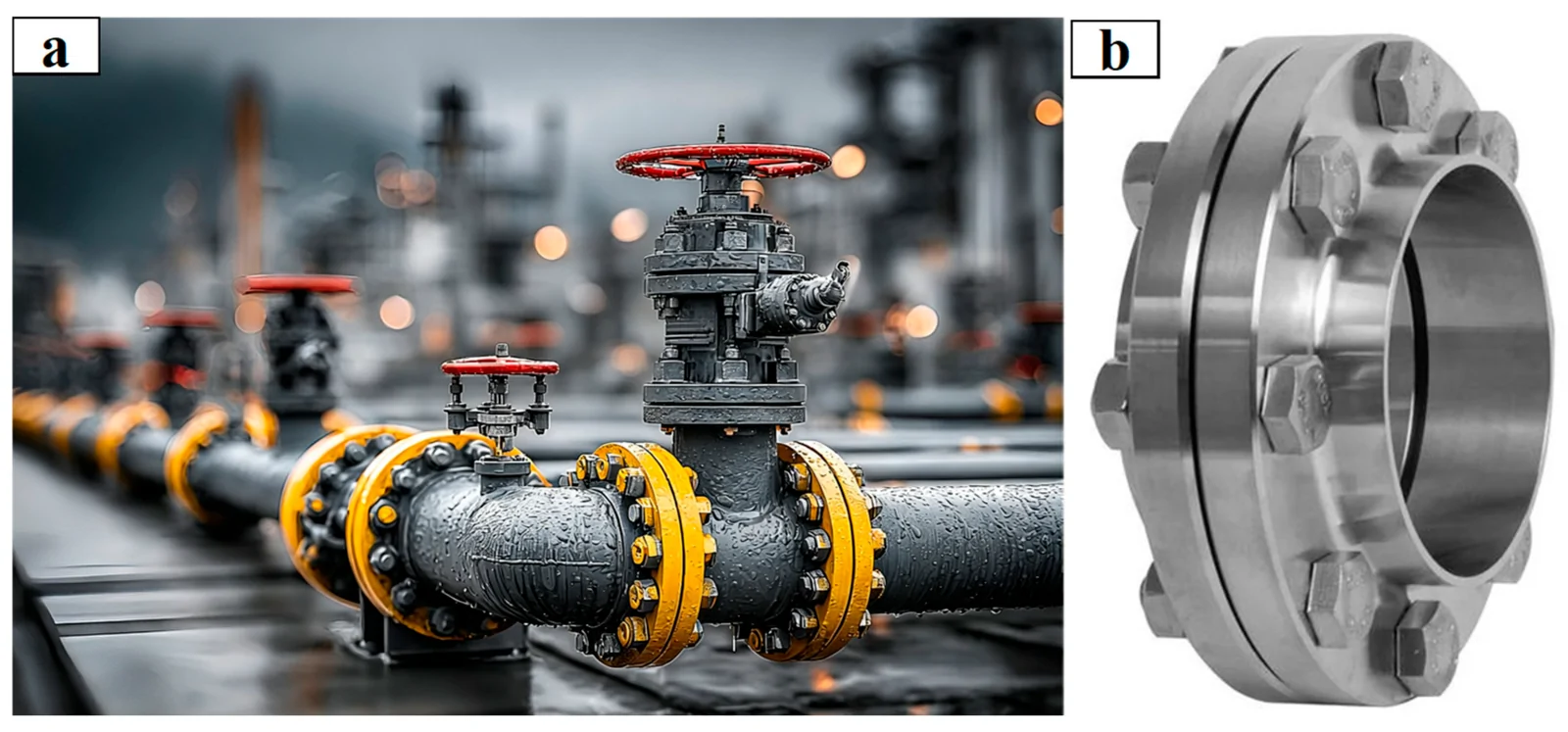
Physical diagram of flange joint: (**a**) Complicated equipment, (**b**) equipment flange.

Flange-bolted joints represent one of the most fundamental connection configurations in complex engineering equipment, as illustrated in Figure 3. Widely deployed across offshore wind power, aerospace, petrochemical processing and general machinery manufacturing [1,2,3], such assemblies offer distinct merits, including straightforward assembly, convenient disassembly and outstanding load-bearing capacity. Nevertheless, the irreversible bolt preload relaxation and structural stiffness degradation persist as pervasive challenges throughout their service life, which have long troubled engineering practitioners. These degradation phenomena undermine joint reliability and load capacity; in extreme scenarios, they may trigger complete structural breakdown and severe safety hazards [4,5].

As critical load-transfer components, flange-bolted connections are indispensable core parts of aero-engines, heavy machinery, high-pressure pipelines and wind turbine towers, valued for their superior sealing performance and structural stability. When subjected to prolonged complex dynamic loads—including cyclic excitation, mechanical impact and fluctuating temperatures—they inevitably undergo progressive mechanical degradation. Such deterioration not only erodes the overall stiffness and structural integrity of an assembly, but also risks catastrophic consequences, such as sealing failure, structural separation and total functional loss [5]. Accordingly, in-depth investigation into the mechanical degradation evolution of flange joints constitutes both a classic fundamental research topic in mechanical connection mechanics and an essential engineering foundation to support long-lifespan, high-reliability structural design and intelligent condition maintenance.

At the same time, the mechanical degradation evolution rules, interface friction constitutive models and multi-bolt coupling degradation mechanisms systematically sorted out in this review provide essential theoretical support for sensing signal demodulation, bolt preload quantitative measurement and long-term flange structural health monitoring. Various sensors, including FBG strain sensors, piezoelectric sensors, eddy current displacement sensors, ultrasonic monitoring devices and acceleration sensors have been widely adopted to capture the degradation signals of bolted flange joints [6,7].

### 1.3. Definition and Classification of Mechanical Degradation of Flange-Bolted Joints

In this work, the mechanical degradation of bolted flange structures is uniformly defined as an irreversible deterioration of interfacial mechanical states and global mechanical performance induced by long-term combined service loads (cyclic vibration, impact, thermal cycling and internal medium pressure). Note that the amplitude-dependent damping variation is a reversible nonlinear dynamic response that does not cause permanent interface damage, and thus is excluded from intrinsic mechanical degradation. All irreversible degradation originates from contact microslip, surface plastic embedding and cumulative elastic–plastic deformation. Two easily confused axial force loss mechanisms are strictly distinguished to eliminate logical ambiguity:

Preload relaxation: Axial clamping force attenuation induced by gasket creep, surface embedding and inter-bolt elastic interaction. No relative rotation occurs between the nuts and bolt threads; a partial clamping force can be recovered via secondary tightening, showing partial reversibility.

Rotational self-loosening: Severe, irreversible preload loss caused by nut–thread relative motion under alternating transverse loads. It is an extreme derivative form of preload relaxation, and the lost preload cannot be recovered through simple re-tightening without thread repair.

Six mutually coupled yet mechanically independent intrinsic interfacial degradation modes are summarized, with distinct generation mechanisms, characterization indexes and evolution paths:

(1) Preload relaxation: The primary precursor of all interfacial degradation, originating from creep, embedding and elastic interaction.

(2) Rotational self-loosening: Severe irreversible preload decay triggered by nut rotation under cyclic transverse excitation.

(3) Stiffness attenuation: Progressive softening of global joint stiffness as interfacial micro-/macroslip expands.

(4) Amplitude-dependent damping variation: Reversible frictional energy change, which is only an auxiliary dynamic feature rather than permanent degradation.

(5) Interfacial wear: Irreversible surface abrasion from reciprocating microslip, which accelerates preload loss and stiffness decay.

(6) Structural fatigue damage: Long-term derivative damage induced by alternating uneven stress under degraded joint stiffness.

The sealing leakage is excluded from the intrinsic interfacial degradation categories. It is a secondary macroscopic engineering failure that only emerges after the preload loss and contact pressure non-uniformity exceed critical thresholds, rather than an independent interfacial evolution mechanism.

Notably, fatigue failure is long-term structural damage deriving from cyclic alternating stress under degraded stiffness and uneven bolt load distribution, which is taken as the derivative failure consequence of mechanical degradation rather than a core degradation form of flange contact interfaces. In the following experimental and theoretical analysis, we strictly distinguish the above six core degradation phenomena and clarify their coupling evolution relations.

## 2. Experimental Studies on Degradation of Flange Connection Structure

Bolt degradation experiments serve as an essential bridge linking theoretical modeling to practical engineering, furnishing irreplaceable measured data for revealing interfacial degradation mechanisms and developing physics-based constitutive models. Mechanical degradation within flange-bolted joints consists of coupled multi-physical processes, including bolt preload relaxation, structural stiffness attenuation and interfacial frictional energy dissipation. Pure analytical derivations alone cannot fully capture the intricate evolutionary behaviors of bolted interfaces. Therefore, systematic and comprehensive experimental investigations are required to extract their characteristic parameters, verify the theoretical frameworks and guide on-site engineering applications. Establishing standardized benchmark specimens and high-precision multi-condition test platforms, and performing multi-working-condition degradation and self-loosening tests, will lay the fundamental groundwork for advancing our fundamental understanding of flange joint mechanical degradation.

Based on the current research findings, the experimental research on the nonlinear dynamics of bolted connections originated in Germany. The Brake–Reuβ beam, double-mass dumbbell device, Gaul resonator, Sumali double beam and other classical standard specimens have been developed. A large number of experiments and benchmarking verifications have been carried out around the interface friction energy dissipation, stiffness and damping nonlinearity, constitutive model parameter identification, and so on. In contrast, although research in China has relied on the introduction of foreign equipment, it has developed rapidly in the past ten years. It has been able to independently develop a variety of testing machines and testing systems with distinct characteristics related to multi-mode loading, high-precision monitoring and standard setting, constructing a relatively complete experimental research system. However, the existing experimental research still faces three deficiencies: First, the prediction accuracy of damping characteristics is much lower than that of frequency prediction, and the overall dynamic modeling error of large equipment with interface nonlinearity is still prominent. Second, most experiments are based on a single bolt or simplified plate structure, which is different from the load transfer and synergistic degradation behavior of real multi-flange-bolted systems. Third, the transfer mechanism of the interface micro-friction behavior to the macro-response is not clear, and the experimental data accumulated under multiple working conditions is still not fine enough. Among these developments, Section 2.1 reviews the classical reference specimens and their main findings, which are suitable for the study of joint interface dynamics; Section 2.2 introduces the working principles and testing abilities of various special testing devices (such as the Junker transverse vibration testing machine, Schatz multi-function fastening analysis system, multi-mode loosening test device, etc.); Section 2.3 focuses on the experimental law of bolt preload relaxation, including the systematic observation results for the influencing factors, such as the tightening process, load conditions and elastic interaction. By combing through the above experimental research, the aim is to clarify the maturity and core bottlenecks of the current experimental system. This will provide a reliable experimental basis for subsequent theoretical modeling and multi-bolt coupling degradation research.

### 2.1. Research on the Standard Experimental Benchmark

#### 2.1.1. Brake–Reuβ Beam Structure

**Figure 4:**
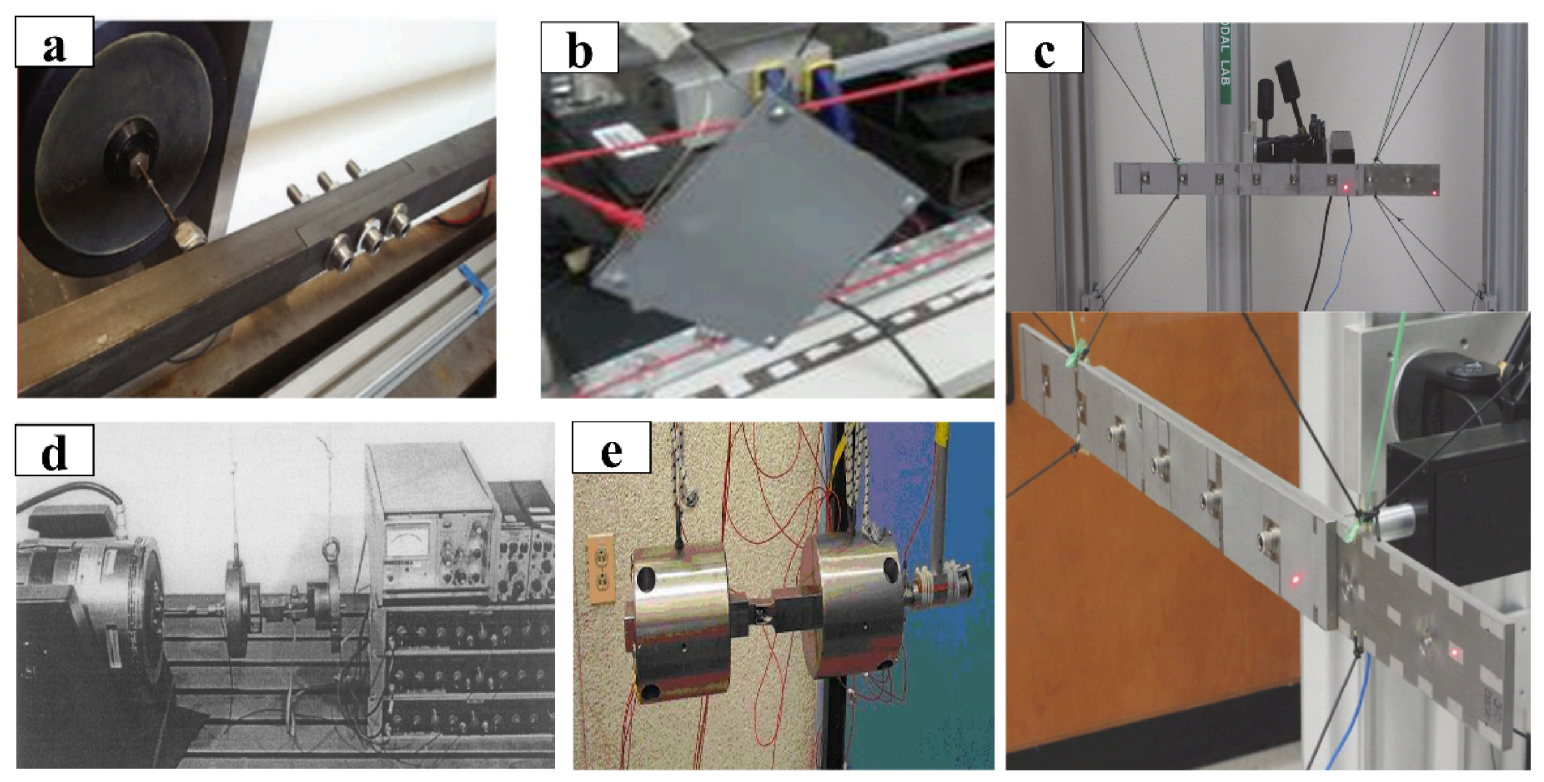
Standard experimental reference object physical map: (**a**) Brake–Reuβ beam structure, (**b**) four-bolt connection square plate, (**c**) Sumali double-beam lap joint structure, (**d**) Gaul resonator, (**e**) dual-mass dumbbell device.

A Brake–Reuβ beam [8,9] is a standardized experimental model for studying the dynamic behavior of friction-containing interface connection structures, which is widely used in mechanical connection, nonlinear vibration and structural dynamics. Its structure is shown in Figure 4a. It usually consists of three parts: an integral beam (without joints), an integral beam with three interfaces (simulated with contact but without bolts), and a beam with three bolted joints (complete Brake–Reuβ model). In the modal test of Brake on the Brake–Reuβ beam, it was found that there were obvious nonlinear characteristics of stiffness and damping. Gross et al. constructed a numerical benchmarking analysis framework [10]. A comparative study was conducted on the three types of modeling methods proposed by Imperial College, the University of Stuttgart and Sandia National Laboratory. Taking the Brake–Reuβ beam as the reference specimen, the prediction effects of structural stiffness and damping nonlinear characteristics were verified, respectively. The applicable scenarios, advantages and limitations of various methods were clarified. Flicek [11] constructed a high-precision finite element model of the Brake–Reuβ beam. The mechanical response law of the structure under an impact load was systematically studied. The effects of key parameters, such as the impact strength, action time, impact position and interface residual stress, on the friction energy dissipation characteristics were analyzed. Lacayo et al. [11] proposed a parameter correction method based on an efficient quasi-static modal algorithm to update the Iwan element constitutive parameters of the Brake–Reuβ beam connection interface in a finite element framework. The results showed that the method can better reflect the modal coupling phenomenon of beam structure under impact response.

The Brake–Reuβ beam benchmark is suitable for modal nonlinear and amplitude-dependent damping tests under small cyclic excitation. However, its simplified lap structure ignores the circumferential flexible deformation and multi-bolt elastic interaction unique to annular flanges, leading to poor engineering adaptability for pressure-bearing flange equipment. Its measurement defects mainly lie in its inability to reproduce bending and internal pressure coupled loads in real complex equipment.

#### 2.1.2. Four-Bolt Connection Square Plate

A four-bolted square plate is a structural member in which two square steel plates are connected by four screws, as shown in Figure 4b. The system has more significant damping characteristics than the integral components of the same size; especially when a bolt washer is not added, the slap effect between the connecting plates will further lead to additional nonlinear behavior. Segalman et al. [12] preliminarily explored the power-law correlation between the damping energy dissipation and the modal displacement of the structure. The results show that the energy dissipation is significantly affected by the location of the excitation. This conclusion has important reference value for the local kinematics modeling of bolted joints.

The four-bolt square plate specimen is suitable for preliminary qualitative research on amplitude-dependent damping and local joint energy dissipation under small vibration excitation. Its major measurement defect lies in the rigid flat-plate structure without a circumferential curved flange deformation, so it cannot reproduce uneven circumferential contact pressure or the inter-bolt elastic coupling effect of annular pressure-bearing flanges. In terms of engineering adaptability, this benchmark only matches simple plate lap joint scenarios, and the measured stiffness and preload attenuation laws cannot be directly transplanted to large-scale equipment flanges with bending and internal pressure loads.

#### 2.1.3. Sumali Double-Beam Lap Joint Structure

The Sumali double-beam lap joint structure is composed of two stainless steel beams of the same size lapped by multiple bolts, as shown in Figure 4c. The experimental model is a classical specimen for studying the nonlinear damping, interface slip and amplitude-dependent dynamic characteristics of bolted joints. Its structure forms an overlapping interface through bolt fastening, and shows obvious stiffness softening, damping enhancement and energy dissipation power-law characteristics under vibration and impact loads. It is often used to verify the accuracy of joint constitutive models, such as Iwan model and Bouc–Wen model. Deaner et al. [13] carried out a systematic analysis of the structure. The results show that each mode of the structure can be effectively approximated by a single-degree-of-freedom system. The nonlinear connection adopts a four-parameter modal Iwan model and is described in parallel with a linear spring and a viscous damper. Numerical and experimental comparisons confirm that the model can accurately characterize the nonlinear stiffness and damping evolution characteristics of the structure within a certain load range.

The Sumali double-beam lap joint is well-suited to investigate amplitude-dependent stiffness softening and frictional energy dissipation under low-to-medium cyclic shear excitation, and it can efficiently calibrate the basic parameters of hysteresis constitutive models, such as the four-parameter Iwan model. However, the parallel straight-beam layout eliminates the circumferential bending and asymmetric pressure distribution featured by annular flanges; moreover, the uniform bolt arrangement cannot reproduce the elastic interaction coupling between bolts on circular flange rings. Therefore, the dynamic degradation laws obtained from this specimen only apply to straight lap plate structures, with poor transferability to the circular flange joints of pressure vessels and power equipment under bending and internal pressure combined loads.

#### 2.1.4. Gaul Resonator and Dual-Mass Dumbbell Device

A Gaul resonator [14] is a classical longitudinal resonance experimental device proposed by Professor Lothar Gaul’s team at the University of Stuttgart in Germany for the study of fretting friction, slip, damping and energy dissipation at a bolted interface. It is one of the landmark experimental platforms in the field of nonlinear dynamics of bolted joints, and is shown in Figure 4d. The dual-mass dumbbell device [15,16] is a classical bolted joint dynamics experimental platform proposed by Sandia National Laboratory. It is also suitable for studying the fretting friction, energy dissipation, stiffness softening and amplitude-dependent damping characteristics of bolt interfaces, and is shown in Figure 4e. Dominik et al. [15] treated the Gaul resonator as equivalent to an equal-degree-of-freedom dynamic model, and used the multi-harmonic balance method to solve and analyze the dynamic response of each equivalent mass in the model. In order to improve the universality of the model, the researchers also constructed a finite element model of the Gaul resonator, and used the zero-thickness element to equivalently treat the bolted joint interface. The numerical results show that the above two modeling methods can accurately simulate the interface friction hysteresis characteristics and the overall dynamic response of the structure. The dual-mass dumbbell device is composed of two concentrated mass blocks in series through a single-bolt/multi-bolt lap joint, and the whole is dumbbell-shaped. A free suspension or soft support boundary is adopted to eliminate the external damping interference. The axial resonance of the structure is excited by impact excitation or harmonic excitation to generate controllable microslip at the interface. Segalman et al. [16] designed and processed bolted joint specimens with various structural forms and carried out test experiments. They systematically expounded the post-processing flow of the acceleration attenuation signal of the dumbbell device under impact excitation, and finally obtained the correlation law of structural damping and energy dissipation with the change in excitation amplitude.

Both the Gaul resonator and dual-mass dumbbell device are classical single-bolt benchmark platforms dedicated to fretting friction, microslip and amplitude-dependent damping tests under longitudinal harmonic excitation, and they are widely adopted to calibrate the core Iwan hysteresis parameters. Nevertheless, two obvious measurement limitations restrict their engineering extension. First, both setups only bear axial reciprocating loads without reproducing the bending moment, internal medium pressure or uneven circumferential contact pressure of annular flanges. Second, they merely contain one or two bolts, which cannot capture the elastic interaction and cross-coupled preload relaxation between multi-bolt groups on circular flange rings. In terms of engineering adaptability, the test conclusions derived from the two devices are only valid for simple single-point lap joints; they cannot be directly used to predict the full-cycle coupled degradation behavior of multi-bolt flanges in aerospace and pressure-bearing complex equipment.

As can be seen from the above, other countries have formed a relatively complete experimental system for the nonlinear dynamics of bolted joints. The Brake–Reuβ beam, double-mass dumbbell device, Gaul resonator, double Sumali beam and other classical standard specimens have been developed successively. Much research has been carried out on interface friction energy dissipation, stiffness and damping nonlinearity, constitutive model parameter identification, multi-algorithm benchmarking verification, and so on. Scholars have continuously optimized the joint simulation method from the perspectives of finite element modeling, zero-thickness interface element, multi-harmonic balance algorithm, and modal parameter update. These works on degradation experiments of bolt connections have created a solid foundation. However, concerning the research status of experimental test benchmarks, there are still the following deficiencies: The prediction accuracy of damping characteristics is much lower than that of frequency prediction. The overall dynamic modeling error of large equipment with interface nonlinearity is still prominent. The transfer mechanism of interface micro-friction behavior to macro-response and the model’s operation under multiple working conditions are not fine enough.

All the above single-/double-bolt benchmark specimens ignore the inter-bolt elastic interaction and circumferential flexible deformation unique to multi-flange-bolted systems, so their measured dynamic laws cannot be directly applied to full-scale flange systems.

### 2.2. Typical Test Device and Test Method

With regards to measuring the hysteresis characteristics of bolted joint interfaces under different conditions, researchers have made systematic and in-depth progress in experimental methods, mechanism exploration and detection technology, in close connection with major engineering needs. Considering the current situation, eight typical experimental devices used at home and abroad are reviewed and analyzed.

#### 2.2.1. Mechanical Lap Joint Fretting Wear Test Device

**Figure 5:**
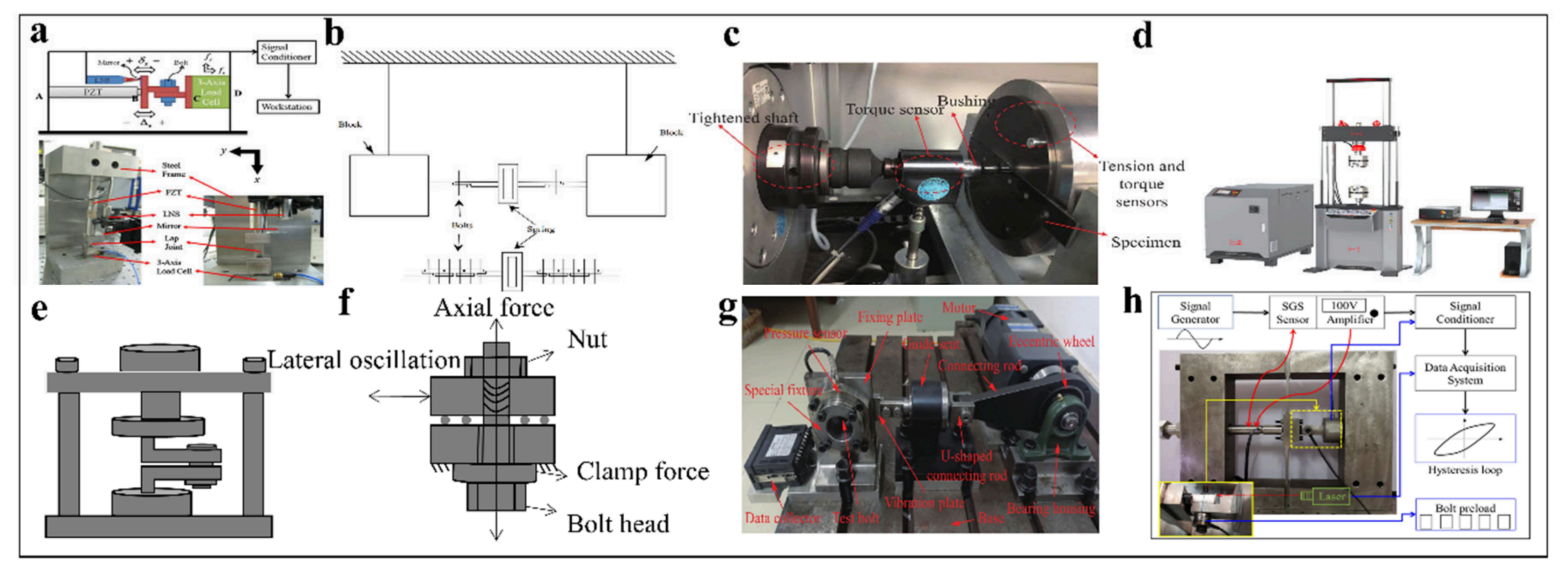
Typical test setups: (**a**) Mechanical lap joint fretting wear test device, (**b**) damping characteristic measuring device, (**c**) Schatz-Analyse multi-functional bolt tightening analysis system, (**d**) universal testing machine, (**e**) multi-mode loosening test device, (**f**) Junker lateral vibration testing machine, (**g**) Junker lateral vibration testing machine, (**h**) a new test device for measuring friction hysteresis.

The fretting wear test device for a mechanical lap joint is a special experimental device developed and designed by M. Erite [17], as shown in Figure 5a. The device uses a piezoelectric actuator to apply a micro-motion displacement, measures the interface tangential force and the additional force generated by the misalignment through a triaxial force sensor, and uses a laser nano-sensor to collect the relative slip of the contact surfaces on both sides of the joint. During the test, the micro-motion response data collected by the equipment do not need additional post-correction, filtering or other processing, as shown by original test results of the integrated specimen, single-bolt aluminum lap joint and steel lap joint under multiple load conditions. The signal noise, installation eccentricity error, additional stiffness and additional damping interference caused by the equipment itself can be ignored, and the device can accurately characterize the real fretting evolution law of mechanical lap joints.

This mechanical lap joint fretting wear test apparatus is specially designed for nanoscale relative slip measurement and quantitative analysis of interface tribological evolution under tiny reciprocating displacements, which is ideal for exploring the micro-mechanism of early preload loss induced by fretting friction. Its prominent measurement defects include an ultra-small stroke limitation and it is incapable of applying large cyclic shear loads, bending moments or internal pressures that real flanges endure. In addition, it only supports single lap specimens without a circular flange ring and multi-bolt layout, failing to reflect the uneven circumferential contact pressure and inter-bolt elastic coupling. Therefore, this equipment is only applicable to microscopic tribology mechanism research rather than full-range degradation tests for engineering annular flanges, with low adaptability to the complex service load conditions of large-scale equipment.

#### 2.2.2. Damping Characteristic Measuring Device

Goyder et al. [18] developed a test device for studying damping characteristics, as shown in Figure 5b. The test device consists of two mass blocks and a spring, which can be excited to generate vibration and apply a load to the bolt connection. Through the vibration attenuation process, the energy dissipation rate can be measured. The subsequent experimental research showed that the damping characteristic measuring device can display the relationship between the number of bolts and damping according to different test bench configurations.

This damping characteristic measuring device is tailored to quantitatively extract the structural damping ratios and energy dissipation laws under free vibration attenuation, and it can conveniently compare the damping contribution of different bolt quantities. However, it can only realize uniaxial vibration excitation, and cannot apply cyclic shear loads, bending moments or internal pressures that real flanges bear during service. Moreover, the simple two-mass–spring layout cannot reproduce the uneven circumferential contact pressure and inter-bolt elastic coupling of annular flange assemblies. Its engineering adaptability is limited: it is only suitable for preliminary qualitative damping comparisons of simple bolted components, and cannot acquire the complete force–displacement hysteresis curves needed for calibrating Iwan-type degradation constitutive models.

#### 2.2.3. Schatz-Analyse Multi-Functional Bolt Fastening Analysis System

The Schatz-Analyse multi-functional bolt fastening analysis system was developed and is produced by Schatz AG, Germany [19,20]. The equipment provides extremely high precision and comprehensive analysis capabilities; with a variety of sensors, it can more accurately measure the total tightening torque, clamping force, angle, and end friction torque [21]. Figure 5c shows a typical test device. The drive unit has a built-in servo motor and a precision reducer, which can apply torque at a high-precision speed of up to 150 rpm. The measurement unit contains multiple high-precision sensors, mainly torque/angle sensors and axial force/thread torque sensors, which can simultaneously measure key parameters, such as total torque, thread torque and axial preload. Fixture unit: It can be used to fix the bolt, nut, gasket and connected parts (such as 45 # steel plate), which can be replaced to simulate different working conditions. Control system: The core is an industrial computer and SCHATZ^®^-ANALYZE special software, which is responsible for controlling the test, collecting data and automatically calculating the torque coefficient, friction coefficient, and so on. Safety protection device: It is equipped with a transparent protective cover, emergency stop button, etc., to ensure safe operation.

From the relevant research literature, it can be seen that the academic community has generally recognized the system: it is, first of all, an accurate measurement tool that meets the ISO16047 standard and has 0.5% high precision [22]; at the same time, it accurately captures multiple parameters and automatically analyzes capabilities, making it a powerful engineering analysis platform.

Nevertheless, this equipment has prominent inherent measurement defects for cyclic degradation research: It lacks long-term reciprocating transverse vibration loading modules, and cannot continuously capture the preload attenuation and interfacial hysteresis responses under thousands of cyclic loads. In addition, its fixture only supports small single-bolt specimens without interchangeable annular flange tooling, making it impossible to reproduce the uneven circumferential contact pressure and multi-bolt elastic coupling effects. In terms of engineering adaptability, this system is only suitable for the static assembly parameter calibration of fasteners; it cannot conduct full-cycle stiffness degradation and preload relaxation tests for engineering flange joints under dynamic alternating loads, and cannot provide the hysteresis loop data required for calibrating time-varying Iwan constitutive models.

#### 2.2.4. Universal Testing Machine

The universal testing machine, also known as the universal material testing machine, is a general standard precision device for quasi-static testing of mechanical properties of materials. It can complete tensile, compression, bending, shear and other mechanical tests. It is the core instrument for the basic mechanical calibration of metal/non-metallic materials and joint specimens in universities and research institutes; a typical test device is shown in Figure 5d [23]. The extensometer is used to measure the small deformation of the high-precision sample gauge section and obtain the real strain data. As the core component, the measurement and control system mainly assures closed-loop control of loading rate, force value and displacement, and automatically collects and generates the force–displacement and stress–strain curves. Ferrero and Abad [24,25] pointed out through experiments that the universal test based on quasi-static conditions is the simplest method to obtain the hysteresis curve of a joint.

The universal testing machine is a versatile quasi-static loading platform that can easily obtain full force–displacement hysteresis loops of bolted joints under controlled shear loads, and serves as the basic test equipment for offline calibration of Iwan model static parameters. Its obvious measurement defects lie in the lack of long-term cyclic vibration excitation modules that can simulate thousands of alternating load cycles that occur in real service, and it cannot synchronously apply the bending moment and internal pressure coupled loads that act on actual annular flanges. Moreover, the standard fixtures only support simple lap specimens and cannot reproduce the uneven circumferential contact pressure or elastic interaction between multiple bolts on flange rings. From the perspective of engineering adaptability, this equipment is only useful for single-cycle quasi-static hysteresis tests and static stiffness measurements, and it cannot carry out long-time continuous monitoring of preload relaxation and progressive stiffness degradation under dynamic cyclic excitation of complex equipment flanges.

#### 2.2.5. Multi-Mode Loosening Test Device

The multi-mode loosening test device is a kind of loosening test device for bolted connection structures developed by Zhu Minhao’s team [26] at Southwest Jiaotong University; a typical test device is shown in Figure 5e. The multi-mode loosening test device can perform a loosening test of bolt connection structure under multi-mode loads (eccentric, torsional, axial alternating and shear alternating). Its structure is simple, easy to operate and is low cost. At the same time, its control and test accuracy is high, and the test data are accurate and reliable. It can easily provide a testing basis for the design and use of bolt connection structure, and so can more effectively improve the reliability of bolt connection structures [26]. In subsequent research, the technical ideas and methods used in the device have been cited many times, which is enough to prove that the device is effective for load experiments.

This self-developed multi-mode loosening test device can realize multi-directional alternating loads, including eccentricity, torsion, axial force and shear force, simultaneously, and it has a low manufacturing cost and flexible fixture replacement, which is suitable for multi-load coupling preload relaxation tests of small simplified flange specimens. However, compared with imported standardized testing equipment, its preload measurement accuracy is slightly lower, and it lacks high-precision synchronous displacement acquisition modules for microslip. More importantly, the existing fixture design cannot simulate large-size annular flanges with circumferential flexible deformation, and it fails to reproduce the uneven contact pressure distribution and cross-coupled preload loss that occurs among multiple circumferentially arranged bolts. In terms of engineering adaptability, this device is only applicable to preliminary mechanism explorations of small bolted samples under composite loads; it cannot provide the high-precision long-cycle hysteresis data required for accurate calibration of time-varying Iwan degradation models of large complex equipment flanges.

#### 2.2.6. Junker Transverse Vibration Testing Machine

The Junker transverse vibration testing machine was first invented by the German engineer Gerhard Junker in the 1960s. After that, Li Haijiang’s team at Tsinghua University [27] added an eddy current displacement sensor to the upper connecting plate of the device. Figure 5f shows a typical test device. It can achieve the synchronous measurement of the clamping force and the lateral displacement of the thread connection during the process of transverse vibration. After this improvement and upgrade, the researchers could reveal the dynamic details of the loosening mechanism more microscopically. Through the Junker lateral vibration testing machine, the research team made two important findings: First, when numerically fitting the clamping force attenuation curve, the initial clamping force drop follows a double exponential function relationship. Second, the loosening speed is mainly determined by the initial preload and the lateral vibration amplitude. As a high-precision and standardized testing tool, the Junker transverse vibration testing machine is widely used in aerospace, automobile manufacturing, wind power, rail transit and other industries with high requirements for connection safety, and has become a benchmark for fastener anti-loosening performance.

The Junker transverse vibration testing machine is the internationally recognized standard bench for studying bolt self-loosening under cyclic lateral excitation, capable of real-time synchronous monitoring of transverse displacement and residual preload with high precision, and its test data can directly calibrate the preload attenuation law for single-bolt specimens. However, it has obvious inherent measurement limitations: It only supports single-bolt fixture structures and cannot assemble annular multi-bolt flange specimens, so the uneven circumferential contact pressure and elastic interaction between adjacent bolts cannot be reproduced. Moreover, it lacks loading modules for the bending moment and internal medium pressure, which are critical service loads for pressure vessel and aero-engine flanges. In terms of engineering adaptability, the test conclusions obtained by this machine are only valid for independent single-bolt lap joints; the preload relaxation and stiffness degradation laws measured cannot be directly extended to multi-bolt flange systems with ring flexible deformation, and it cannot acquire complete long-cycle hysteresis curves for time-varying Iwan model full-parameter identification.

#### 2.2.7. Displacement Loading Transverse Vibration Test System

The displacement loading transverse vibration test system was developed and designed by Lin Zhigen’s team at Nanchang Hangkong University [28]; Figure 5g shows a typical test device. The system is driven by a variable frequency speed regulation motor, and transmits power through the center crank-slider mechanism. In the vibration test, the change in preload is collected in real time by an RDF-K108 pressure sensor, and the signal is uploaded to the computer for analysis and processing. Based on the experimental system, the research team found the characteristics of the stages of the preload relaxation process and the quantitative influence of vibration displacement, initial preload, pitch and other parameters on the preload relaxation rate. It was revealed that the loosening process of the bolt is not nonlinear under transverse vibration, but is clearly divided into three stages: linear loosening, rapid loosening, and complete loosening.

This displacement-controlled transverse vibration test system adopts a crank-slider driving structure, which can stably output an adjustable reciprocating lateral displacement and clearly divide the preload relaxation into three typical evolutionary stages, making it suitable for qualitative research on the staged relaxation law of single-bolt specimens under long-cycle vibration. Nevertheless, the system has prominent measurement and structural limitations: Its displacement driving accuracy is inferior to servo-based Junker equipment, and it lacks integrated modules to apply bending moment and internal pressure loads. Meanwhile, the fixture design only accommodates simple single-bolt lap plates without the capacity to assemble annular multi-bolt flanges, so the uneven circumferential contact pressure and inter-bolt elastic coupling degradation cannot be reproduced. In terms of engineering adaptability, the measured preload evolution data can only reflect the loosening characteristics of small single-bolt samples, and cannot provide the complete force–displacement hysteresis data required for full-parameter calibration of time-varying Iwan models for large engineering flange assemblies.

In addition, Li et al. [29] designed a new test device for measuring friction hysteresis based on the experimental results of the mechanical behavior of bolted joints, as shown in Figure 5h. The device uses a piezoelectric actuator to provide a contact interface with a stable oscillating relative displacement. At the same time, the interface friction force and bolt preload are collected in real time by the force sensor and gasket, and the relative displacement data for the connection interface are obtained by a single laser vibrometer. The research shows that the device can continuously measure the friction force and bolt preload.

Based on the literature review, it was found that there are many new experimental devices for studying bolt connection characteristics. But in general, the existing research focuses on the core issue of bolted connection degradation and constructing an experimental research system of benchmark specimens and special devices. A performance comparison of the bolted connection experimental setups is shown in Table 1. It clarifies the influence of factors, such as the preload force and excitation amplitude, on the stiffness, damping, friction hysteresis and other characteristics of bolted connections. Various modeling methods and parameter correction strategies have been proposed, which provide important experimental and theoretical support for determining the loosening mechanism and state identification of bolted connections.

For measuring the preload attenuation, stiffness degradation and preload relaxation signals in the above testing facilities, four mainstream sensing techniques are widely adopted: fiber Bragg grating (FBG) strain sensors, piezoelectric vibration sensors, eddy current displacement sensors and acceleration sensors [30,31,32]. FBG sensors achieve high-precision continuous preload monitoring yet require embedded installation; piezoelectric sensors capture microslip vibration signals sensitively but are only applicable to dynamic excitation tests; eddy current sensors can realize non-contact displacement measurement, while they fail to acquire the axial clamping force synchronously [33,34,35].

The testing equipment used in a Junker machine and Schatz analysis system has high measurement precision and complete standardized calibration specifications, but can only support single-bolt transverse loosening tests and lack the function to simulate flange bending, internal pressure and multi-bolt coordinated loading. Domestic self-developed multi-mode loosening test devices can realize multi-directional composite loading, yet the overall measurement accuracy and standardized test protocols still lag behind those of imported equipment. Most existing commercial single-bolt test benches cannot simultaneously reproduce the coupling effect of gasket creep, temperature cycle and multi-bolt elastic interaction.

### 2.3. Experimental Rules of Bolt Preload Relaxation

There are various causes of bolt pre-tightening relaxation, including gasket creep, embedded relaxation, elastic interaction, vibration loosening, stress relaxation, etc. The in-depth exploration of the experimental rules of bolt preload relaxation and the quantitative revelation of its evolution mechanism and influencing factors are important prerequisites for optimizing the bolt tightening process and improving the service reliability of connection systems. As the core theoretical basis for mechanical analysis of bolted flange connection systems, the deformation coordination equation provides key theoretical support for the quantitative study of preload relaxation characteristics. The elastic interaction is one of the core causes of preload loss. The experimental observation and model construction of its action rules have important engineering and academic value for accurately predicting the degradation process of preload force over time and for ensuring the sealing performance of connection systems [36]. Based on the main causes of bolt pre-tightening force relaxation, this section focuses on the experimental research progress related to the deformation coordination equation and gasket creep relaxation, systematically reviews the experimental observation results and research evolution of elastic interaction, integrates the existing experimental rules, and provides a reliable experimental basis and theoretical support for the precise control of pre-tightening force and prediction of degradation characteristics of multi-flange-bolted systems.

#### 2.3.1. Classic Analytical Design Theory of Bolt Pre-Tensioned Connections

The bolt pre-tightening connection is the primary load-bearing form of detachable mechanical structures. Friction-type bolts generate frictional resistance at the contact surfaces through the pre-tensioning force, thereby transmitting lateral loads. The pre-tension amount directly determines the connection stiffness, resistance to separation, and fatigue life. Its classic analytical design framework has evolved into a standardized theoretical system. The relevant fundamental computational models are systematically elaborated in several authoritative monographs on mechanical components.

Budynas et al. comprehensively established a fundamental mechanical model for pre-tightened bolt connections in Section 8, on threaded fasteners and non-permanent joints, of *Shigley’s Mechanical Engineering Design (11th Edition, SI Units)*. This is the general design reference theory applicable to both flange-sealed bolts and conventional friction bolts. Section 10 of the sixth edition of Juvinall and Marshek’s *Fundamentals of Mechanical Component Design* adopts the Shigley dual-spring stiffness core framework. They simplified the engineering calculation steps: focus on the overall stress equilibrium design of bolt groups on the pressure vessel flanges, then propose a design approach for compensating for the non-uniform preload in multi-flange-bolted systems. Hall et al. established the paradigm for the deformation equilibrium analysis of pre-tensioned connections in Section 13 of *Machine Design* at an earlier stage. It serves as the theoretical foundation for all subsequent monographs on threaded connections, and together with the other two components, they form a comprehensive theoretical framework for the traditional analytical design of bolted pre-tightened connections. The traditional analytical design method offers the advantages of simple calculations and strong engineering adaptability, making it widely applicable to the design of conventional flanges and mechanical base bolts. The limitation lies in the model’s reliance on a homogeneous elasticity assumption, which fails to account for nonlinear degradation behaviors, such as gasket creep, microscopic slip at contact surfaces, and pre-tightening relaxation. This constitutes the fundamental premise of our study on mechanical degradation testing and nonlinear modeling of flange connections.

Flange–bolt connections are the most widely used in the fields of petroleum, chemical engineering, and pressurized oil and gas equipment. Their global engineering design, manufacturing, and assembly processes strictly adhere to a comprehensive set of standardized specifications. The core standards impose mandatory requirements regarding flange dimensions, bolt material selection, pre-tightening control, and seal verification. Appendix 2 of ASME BPVC VIII-1 “Code for Pressure Vessels” [37] provides the complete strength calculation rules for annular gasket flanges. Define the minimum assembly tightening stress y and the operational seal ratio pressure coefficient m for a gasket. Determine the total bolt preload required for the flange based on medium pressure and the gasket material, and specify the appropriate bolt material grade for high-temperature and high-pressure applications (e.g., A193B7 chromium–molybdenum steel). This is the mandatory standard for the design of pressure-bearing flange foundations. The ASME PCC-1 “Guidelines for the Assembly of Pressure Flange Bolts” directly employs Shigley’s torque-to-pre-tension conversion model. Standardize the step-by-step tightening procedure for flanges, adjust the torque coefficient accordingly, and specify the requirements for pre-tightening and re-tightening under high-temperature conditions. Address the key engineering challenges, such as inconsistent pre-tension forces during on-site assembly and failure of gasket seals. Compliant with ASME B16.5/B16.47 standards, standardize the number of flange bolts and diameter arrangement specifications for different pressure ratings. The API pipeline specification addresses the high-temperature, variable-pressure flanges used in oil and gas transportation, incorporating additional verification criteria for pre-tightening relaxation under creep conditions. It establishes an upper limit for bolt pre-tension stress in low-temperature LNG equipment, creating cross-industry complementary constraints with the ASME Pressure Vessel Standard.

All existing international standards are formulated based on the traditional elastic steady-state analytical model. Only the static strength of flanges and bolts, and the minimum pre-tension requirements for sealing have been verified. The patterns of pre-tension force attenuation and connection stiffness degradation caused by gasket creep and contact surface wear under long-term service have not been quantified. Conducting experiments and modeling on the mechanical degradation mechanisms of flange–bolt connections can complement and improve existing standard steady-state design methods.

#### 2.3.2. Experimental Study on Deformation Coordination Equation and Gasket Creep Relaxation

The deformation coordination equation is the theoretical cornerstone of the analysis of bolt–flange connection systems. Its core idea is that under the action of external loads, the elongation change in the bolt and the compression change in the flange and gasket are geometrically coordinated with each other. This idea was initially established in the 1990s. Bibel and Ezell discovered, through elastic interaction experiments on pipeline flanges, that adjacent bolts will cause the pre-tightening force of tightened bolts to significantly decrease during the tightening process [38].

In high-temperature environments, gasket creep and bolt stress relaxation require time-dependent effects to be included in the deformation coordination equation. Bickford et al. conducted a preliminary evaluation of the behavior of bolted flange connections at high temperatures. Hao Zhengquan et al. [39] derived the deformation coordination equation of flange connections at high temperatures through experimental research on the compression rebound characteristics, stress relaxation characteristics and basic sealing characteristics of flexible graphite composite gaskets at high temperatures. On this basis, the team further carried out a tightness analysis of the system and discussed the prediction and control of leakage in engineering applications. Afterwards, the researchers used similar ideas to derive the high-temperature flange joint deformation coordination equation containing the effects of external bending moment and creep, and verified the applicability of the modified equivalent axial force method to characterizing the nonlinear distribution of gasket stress.

In addition, some studies have established an analysis framework that is more consistent with engineering reality by simultaneously considering the creep of bolts, flanges and gaskets in the deformation coordination equation, and verified the accuracy of the mathematical model of the deformation process based on the elastic–plastic rheology theory through quasi-static deformation experimental parameter measurements of full-size flange specimens [40].

#### 2.3.3. Experimental Observations of Elastic Interactions

Nassar and Alkelani’s experimental research systematically measured the effects of gasket material, gasket thickness, bolt spacing and other factors on the preload loss caused by elastic interaction, and found that gasket creep relaxation plays an important role in the load loss of sealed joints. Nassar and Yang proposed an analytical model of bolt elastic interaction in gasketed flange joints; quantitatively predicted the impact of a single bolt tightening on the pre-tightening force of adjacent tightened bolts; and evaluated the influence of parameters, such as the tightening sequence, gasket elastic modulus, gasket thickness, and clamping length. In order to verify the model, the researchers built a special experimental device and testing process, and achieved good consistency between the analysis results and experimental data [41]. Nenad et al. [42] systematically studied the influence of elastic interaction stiffness on changes in the bolt pre-tightening force by establishing an analytical stiffness model based on the stress distribution, and verified the validity of the prediction results through a finite element simulation and experimental devices. This study proposed, for the first time, the important phenomenon of “the existence of a singular point in the elastic interaction strength in the bolt spacing direction”—at a specific position between two bolts, the elastic interaction approximately disappears. This finding has reference value for understanding the load transfer mechanism of multi-flange-bolted systems. In addition, the team established a generalized mathematical model to evaluate the residual pre-tightening force of bolts based on the deformation compatibility equation and the elastic interaction stiffness, and developed corresponding experimental devices and test procedures to analyze the effects of initial tightening torque, bolt-hole spacing, bolt material, and gasket material on the elastic interaction stiffness. Validation tests carried out on six types of multi-bolt connectors confirmed the good consistency between the model and the experiment [40].

Based on the above experimental research, it can be seen that the deformation coordination equation lays the theoretical framework for the analysis of flange-bolted preload force, and Hao Zhengquan et al. extended it to high-temperature creep conditions through high-temperature performance experiments on gaskets. The experimental observation of elastic interaction started from the early discovery of Bibel–Ezell, through the modeling and experimental verification of Nassar–Yang, and then to the quantitative analysis of stiffness by Wang et al., forming a complete evolutionary chain from phenomenon recognition to quantitative prediction. These experimental rules provide the key experimental basis for preload control and degradation prediction of multi-flange-bolted systems.

The existing experimental rules are mostly derived from single-bolt simplified specimens, and the quantitative formulas of elastic interaction and gasket creep relaxation obtained under single-bolt conditions cannot be directly extended to multi-flange-bolted systems. Most experimental studies only measure the change in the preload, but fail to synchronously capture the coupled evolution of stiffness and damping, resulting in an incomplete experimental characterization of full-process mechanical degradation.

## 3. Bolt Connection Interface Mechanics Model and Stiffness Degradation Theory

In a flange-bolted connection structure of complex equipment systems, friction is a basic and complex physical phenomenon that exists between the contact interfaces. The nonlinear transfer characteristics of the friction interface are often the most important damping mechanism in composite structures [43]. It is also a key factor affecting the prediction accuracy of structural dynamic characteristics, control performance and service reliability. Due to the strong nonlinearity, hysteresis and sensitivity of friction behavior to multiple physical fields (load, speed, temperature, surface state), establishing a mathematical model that can accurately describe its dynamic response has always been one of the core challenges in the fields of mechanical dynamics, control engineering and precision manufacturing. From the earliest Coulomb’s law to today’s dynamic models that can simulate complex behaviors, such as microscopic slip and pre-sliding hysteresis, the research on friction models has undergone a development process from phenomenon description to mechanism revelation, from static simplification to dynamic refinement.

### 3.1. Unified Classification Criterion of Joint Interface Constitutive Models

Various constitutive models have been proposed to characterize the nonlinear tangential response of bolted joint interfaces, with divergent physical assumptions, mathematical formulations and applicable working conditions. To resolve the classification confusion in the existing literature, this subsection establishes a unified classification criterion based on three core features: whether the model describes preslip hysteresis, incorporates velocity-dependent friction effects, or adopts macroscopic order-reduction processing. Four mutually exclusive model categories are defined: static friction models, velocity-dependent dynamic friction models, hysteresis stick–slip microslip models, and reduced lumped joint equivalent models. Their core characteristics are elaborated separately below. Static friction models only describe steady macroscopic sliding without preslip nonlinearity. Dynamic friction models introduce internal state variables to capture rate-dependent friction phenomena, such as the Stribeck effect. Although the Dahl model can reproduce simple preslip deformation, its core governing variable is friction velocity, fundamentally distinguishing it from hysteresis stick–slip models represented by the Iwan and Valanis frameworks, which prioritize cyclic partial microslip without relying on velocity as the primary input. Reduced-order models sacrifice detailed interfacial physical processes for computational efficiency via lumped stiffness–damping macroscopic expressions.

In this review, friction and joint models are categorized based on the modeling scale, physical description capability and loading characteristics. Static friction models mainly describe steady-state friction forces under a constant sliding velocity, and are incapable of capturing the preslip nonlinearity. Dynamic friction models characterize velocity-dependent friction evolution during continuous sliding. Hysteresis models (e.g., Iwan, Valanis) focus on stick–slip behavior and hysteretic energy dissipation under cyclic excitation, which is critical for bolted joint dynamics. Different from the above interface-level constitutive models, reduced-order joint models aim to construct simplified macroscopic stiffness-–damping expressions for whole connections to improve computational efficiency, which can be derived from hysteresis models via order reduction. Notably, cross-category derivation exists among these model groups, and such inclusion relations are easily ignored in the existing literature.

#### 3.1.1. Static Friction Model

Static friction models describe the friction force only for steady macroscopic sliding status. These models do not contain preslip microslip hysteresis behavior; the friction force is mostly expressed as a constant value related to the normal pressure and friction coefficient. They are suitable for scenarios where only full-sliding conditions occur, while they cannot reproduce stiffness softening or energy dissipation before the macroscopic slip. Typical representatives include the Coulomb friction model.

Static friction models mainly describe the friction behavior when the relative speed is zero or close to zero. They are characterized by a relatively simple model structure and few parameters, but there is often a discontinuity when the speed crosses zero.

1. Coulomb model

The Coulomb model is the most basic idealized model to describe friction. It believes that the sliding friction force is proportional to the normal load, and the direction is opposite to the relative speed, and has nothing to do with the speed and contact area. Its expression is

(1) $$F_{f}=m\;N\;\cdot \;s\;g\;n\;(v)$$

Among these, $F_{f}$ is the friction force, *v* is the friction coefficient, *N* is the normal force, and *v* is the relative speed. This model is simple in form, but it cannot describe the “pre-slip” phenomenon from stationary to sliding or complex friction behavior at low speeds.

2. Coulomb Sticky model

With the development of fluid mechanics, people discovered that some fluids are viscous, so the Coulomb viscosity model was proposed. Dahl et al. [44] proposed a mechanical vibration solid friction damping theory. It was found that the viscous friction force comes from the viscosity of the fluid lubricating layer between the contact surfaces, and it is proportional to the speed. When the speed is zero, it is also equal to zero. Its expression is

(2) $$F_{f}=f_{v}\cdot v$$

Among these, $f_{v}$ is the viscous friction coefficient.

Later, in order to make the model more consistent with the experimental data, a model with a nonlinear relationship with the absolute value of velocity was proposed [45]:

(3) $$F_{f}=f_{v}{\left|v\right|}^{\delta_{v}}\cdot \mathrm{sgn}(v)$$

The value of $\delta^{v}$ here depends on the shape of its contact surface.

Therefore, the Coulomb viscosity model is the Coulomb friction plus the viscous friction, written as

(4) $$F_{f}=f_{v}\cdot v+f_{c}\cdot \mathrm{sgn}(v)$$

In the formula, $f_{c}$ is the Coulomb friction force.

3. Static friction–Coulomb–viscous friction model

The static friction model describes the friction force when two objects do not move relative to each other, but have a tendency to move relative to each other [44]. It is related to the size of the external force received and has nothing to do with the speed. If the external force is less than the maximum static friction force, the static friction force is equal to the external force and in the opposite direction; if the external force exceeds the maximum static friction force, the static friction force is equal to the maximum static friction force. Its model is

(5) $$F_{f}=\left\{\begin{array}{cc} \;f_{e} & \;f_{v}=0\;and\;\left|f_{e}\right|<f_{s}\\ \;f_{s}\cdot \mathrm{sgn}(v) & \;f_{v}=0\;and\;\left|f_{e}\right|\geq f_{s} \end{array}\right.$$

In the formula, $f_{e}$ is the external force and $f_{s}$ is the maximum static friction force.

Morin [46] systematically explored the friction characteristics of stationary contact surfaces, and experimentally verified the conclusion that the static friction is significantly greater than the kinetic friction, and both follow the same friction law, clearly distinguishing static friction from kinetic friction for the first time. Static friction is added to the Coulomb viscosity model, and the model becomes composed of three parts: the static friction, kinetic friction and viscous friction. Its model is

(6) $$F_{f}=\left\{\begin{array}{l} \begin{array}{cc} f_{e}, & v=0,\left|f_{e}\right|\leq f_{s}\\ f_{s}\cdot \mathrm{sgn}(f_{e}), & v=0,\left|f_{e}\right|>f_{s} \end{array}\\ \begin{array}{cc} f_{c}\cdot \mathrm{sgn}(v)+cv, & \;v\neq 0 \end{array} \end{array}\right.$$

In the formula, *c* is the viscous damping coefficient.

4. Stribeck model

In 1902, Stribeck [47] conducted a large number of friction tests and found that the friction evolution law of the lubricating interface was significantly different from the linear change characteristics of the traditional Coulomb–viscous friction model: as the relative sliding speed increases, the friction coefficient first decreases rapidly, reaches a minimum value, and then slowly increases, showing typical nonlinear change characteristics. In 1982, Bo and Pavelescu [48] described this phenomenon using an exponential model. Its model is

(7) $$F_{f}=f_{c}+(f_{s}-f_{c})e^{-{\left(\frac{v}{v_{s}}\right)}^{\delta }}$$

In the formula, *v* is the Stribeck velocity, and $v_{s}$ and δ are empirical constants.

**Figure 6:**
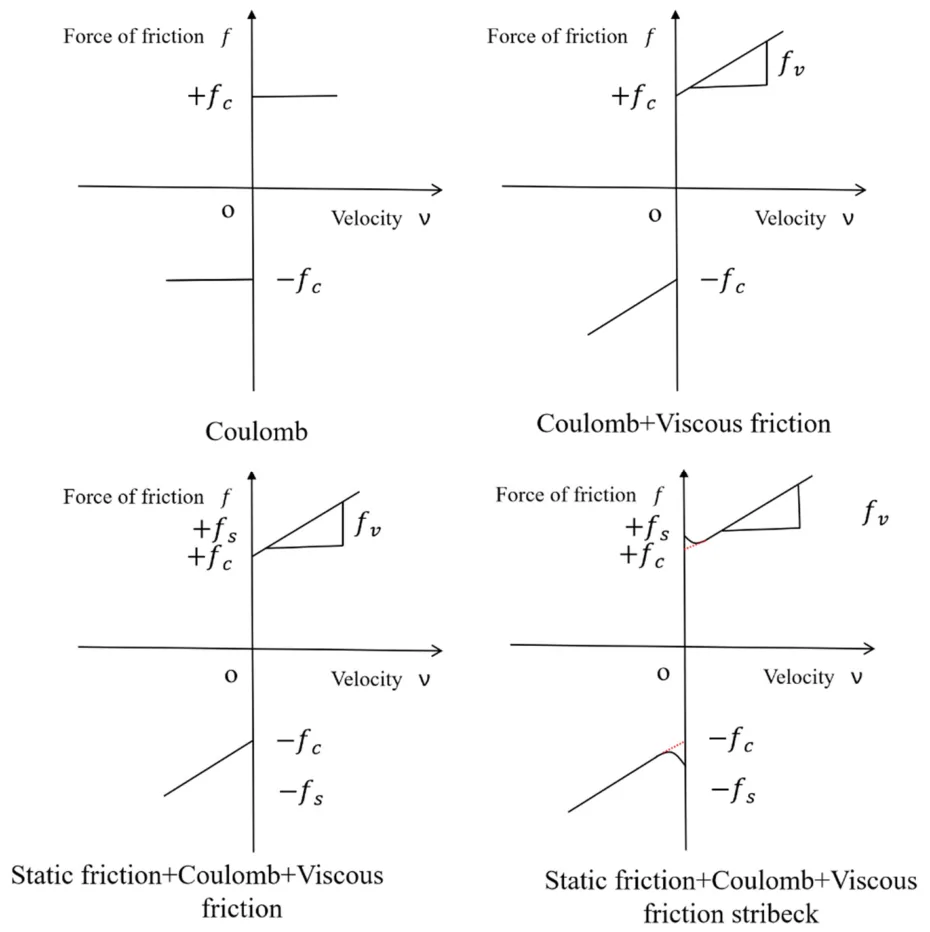
Four static friction models.

Armstrong [49] showed, through systematic experimental research, that the classic Coulomb–viscous model cannot describe dynamic friction problems, such as low-speed nonlinearity, hysteresis, and preslip. Based on this problem, he added the viscous friction term to the Stribeck model to form a more complete Stribeck model. The four static friction models are shown in Figure 6.

(8) f(v)=feif v=0 and fe<fsfs⋅sgn(v)if v=0 and fe≥fsfc+(fs−fc)e−vvsδ+fv⋅votherwise

5. Other models

Karnopp [50] pointed out that stick–slip friction is ubiquitous in various types of actuators and mechanical structures and is a key factor restricting the motion performance of a system. To that end, the author set a zero-speed interval. As long as the speed is within this interval, the speed is considered to be zero, and the friction force is determined by external forces. It is a simple Karnopp model that can directly represent the true zero-speed viscous state. This avoids the problem of judging whether the speed is strictly zero [51], but the size of this interval is difficult to determine.

Armstrong and Dupont [52] proposed a seven-parameter model, which can distinguish the three stages of viscosity, sliding and variable static friction, and describe them very accurately. However, because there are too many parameters, they are difficult to identify and it is difficult to apply them in practice.

Static/dynamic friction models concentrate on the contact friction force, while hysteresis models describe the coupled force–displacement loop; reduced-order models pursue macroscopic equivalence for system-level simulation.

#### 3.1.2. Dynamic Friction Models

Dynamic friction models focus on velocity-dependent friction evolution. State variables are introduced to capture rate-related friction phenomena, such as the Stribeck effect. It should be emphasized that although the Dahl model can describe the pre-displacement effect, its core driving variable is the friction velocity. Typical representatives include the Dahl, LuGre and Leuven models. This category is fundamentally different from microslip-dominated hysteresis stick–slip models.

The study of the dynamic friction model began with Stribeck’s [47] experimental discovery of the dependence of friction speed in 1902. In the 1970s, Dahl proposed a dynamic friction model with internal states, marking the official beginning of dynamic friction modeling. There are many kinds of dynamic friction models, such as the Dahl model, sideburn model, LuGre model, Valanis model, Iwan model, etc. Among these models, the Iwan model has received more attention and research. This type of model can better describe the friction behavior of objects in actual motion and make up for the shortcomings of the static friction model, but it also has problems, such as too many parameters and complex structures.

1. Dahl model

Dahl [53,54] found that before an object reaches the maximum static friction, although it seems not to be moving, it actually has a slight slip, which is called “pre-slip”. The classical Dahl model can reproduce preslip hysteresis under cyclic excitation; nevertheless, it lacks the Stribeck velocity–friction term and cannot account for rate-dependent friction effects. The Dahl model is continuous, which solves the problems of discontinuity and no lag in the static friction model. Its differential equation is

(9) $$\frac{df}{dx}=\sigma_{0}{\left[1-\frac{f}{f_{c}}\cdot \mathrm{sgn}(v)\right]}^{\alpha }$$

**Figure 7:**
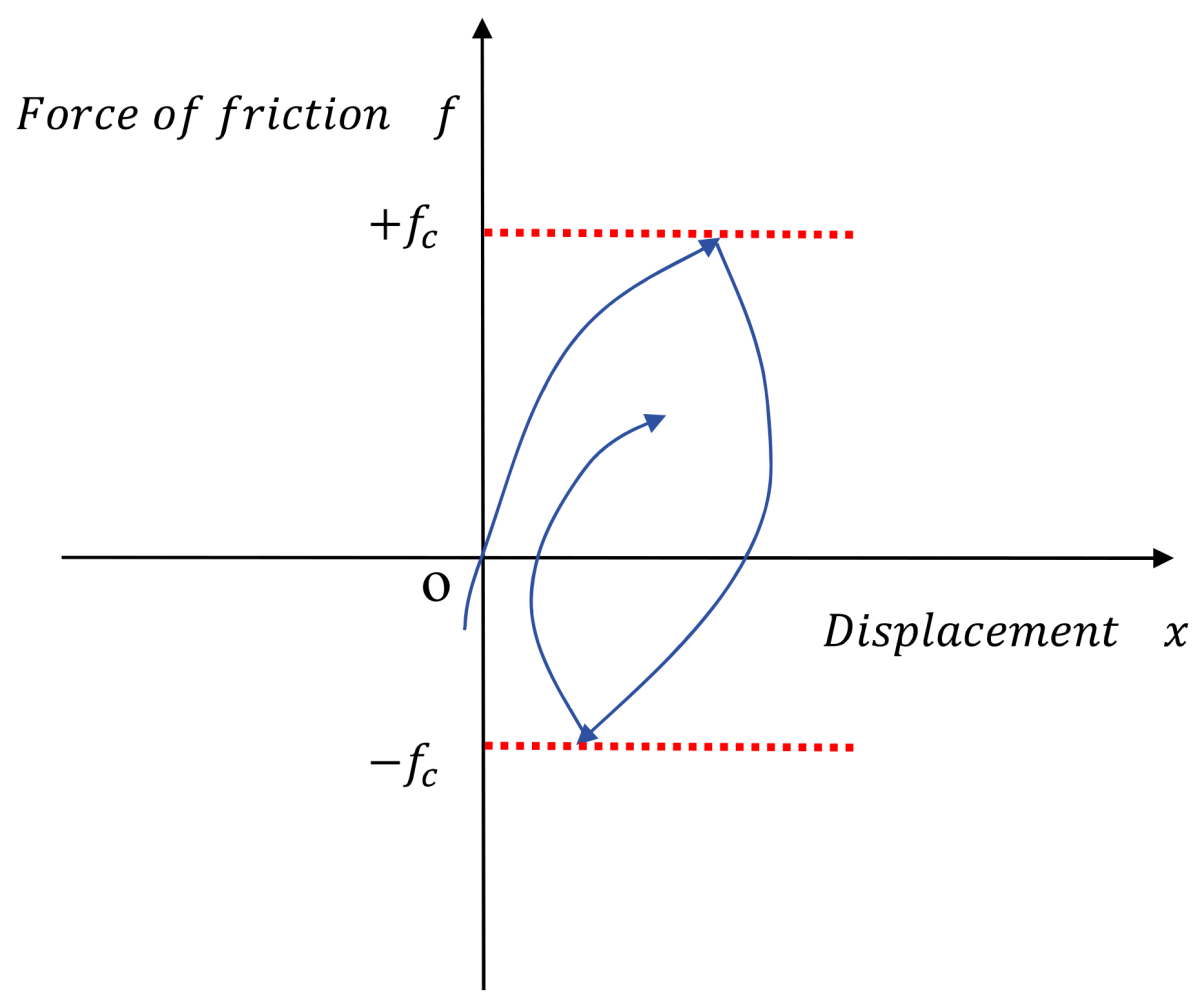
Dahl model.

In the formula, $\sigma_{0}$ is the stiffness coefficient, *x* is the displacement, and α is the fitting parameter. The model diagram is shown in Figure 7.

It can be described in the time domain as

(10) $$\frac{df}{dt}=\frac{df}{dx}\frac{dx}{dt}=\frac{df}{dx}\upsilon =\sigma_{0}{\left(1-\frac{f}{f_{c}}\mathrm{sgn}\left(\upsilon \right)\right)}^{\alpha }\upsilon$$

In the formula, υ is the speed.

2. Bristle friction model

**Figure 8:**
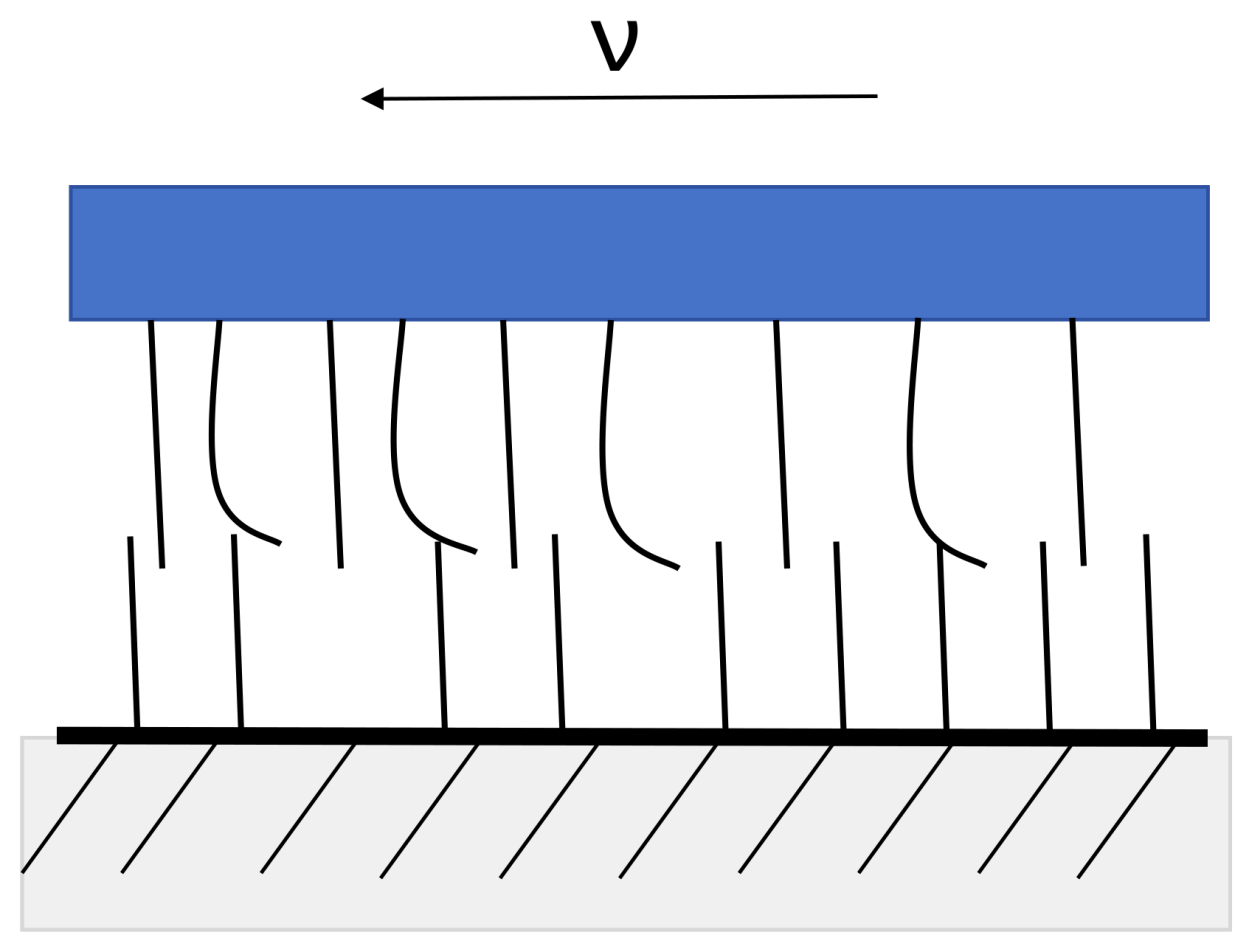
Bristle friction model.

From a microscopic perspective, this model imagines the contact surface as many tiny “hairs” touching and bending with each other, as shown in Figure 8. The interaction between the moving elastic hairs on the upper side and the stationary rigid hairs on the lower side creates friction [55,56]. The greater the number of bristles, the more accurate the calculation is, but it is also more complicated. Generally, the simulation effect is better using 20–25 bristles [57], but due to the complexity of calculation, it is not commonly used in simulations. Its expression is

(11) $$f_{\upsilon }=\sum_{b=1}^{N}k_{0}\left(X_{E,b}-X_{R,b}\right)$$

In the formula, $k_{0}$ is the number of elastic bristles above; $X_{E,b},X_{R,b}$ are the positions of elastic bristles and rigid sideburns, respectively; and *N* is the total number of bristles.

3. LuGre model

The classical Dahl model is capable of reproducing preslip hysteresis behavior under cyclic excitation, whereas it does not incorporate the Stribeck velocity–friction term or rate-dependent friction–velocity characteristics [58]. On this basis, the LuGre model further introduces state-variable-based velocity-dependent friction description and realizes the representation of the Stribeck effect, which is its core improvement over the Dahl model, and has become the mainstream dynamic friction model that simultaneously accounts for both preslip hysteresis and velocity-related friction effects [56]. Limited by its intrinsic mathematical structure, the Dahl model cannot capture velocity-strengthening/weakening friction phenomena, which restricts its application scenarios to bolted flange joints under wide-range variable-speed excitation [54]. Its model function is

(12) $$\frac{dz}{dt}=\upsilon -\frac{z\left|\upsilon \right|}{g\left(\upsilon \right)}\sigma_{0}$$

In the formula, *z* is the average deformation of the sideburns, $\sigma_{0}$ is the stiffness of the sideburns, and $g\left(\upsilon \right)$ is the Stribeck effect. The expression for the friction is

(13) $$f_{\upsilon }=\sigma_{0}z+\sigma_{1}\frac{dz}{dt}+\sigma_{2}\upsilon$$

In the formula, $\sigma_{1}$ is the microscopic damping coefficient (generally a constant [57]), and $\sigma_{2}$ is the viscous friction coefficient.

4. Leuven model

The Leuven model [59,60] (also known as the Leuven integrated friction model) is a dynamic friction model proposed by the team of Jan Swevers, Vincent Lampaert, and Farid Al-Bender of the Department of Mechanical Engineering at the University of Leuven (KU Leuven) in Belgium, from the late 1990s to 2002. The LuGre model has the disadvantages of asymmetric hysteretic curves and insufficient accuracy in the pre-sliding stage, and numerical rigidity and easy divergence when the velocity is reversed. The Jan Swevers team proposed the Leuven model, which can accurately describe sliding and pre-sliding states and is often used in robot control to achieve accurate tracking. Its model is as follows:

(14) $$f_{\upsilon }=F_{h}\left(z\right)+\sigma_{1}\frac{dz}{dt}+\sigma_{2}\upsilon$$

(15) $$F_{h}\left(z\right)=F_{d}\left(z\right)+F_{b}$$

(16) $$\frac{dz}{dt}=\upsilon \left[1-\mathrm{sgn}\left(\frac{F_{d}\left(z\right)}{S\left(\upsilon \right)-F_{b}}\right)*{\left|\frac{F_{d}\left(z\right)}{S\left(\upsilon \right)-F_{b}}\right|}^{n}\right]$$

In the formula, $F_{h}\left(z\right)$ is the static nonlinear force containing non-local memory, $F_{d}\left(z\right)$ is the transition curve, $F_{b}$ is the starting position, $S\left(\upsilon \right)$ is the friction behavior, and *n* is the friction coefficient.

However, this model has six parameters and three mechanisms, which is relatively complex and the parameters are difficult to identify.

#### 3.1.3. Hysteresis Stick–Slip Models

Hysteresis stick–slip models are dominated by cyclic partial microslip behavior under a cyclic tangential load. Their core characteristic is to reproduce the stick-to-slip transition, tangential stiffness degradation and closed hysteresis loops under cyclic excitation, without taking the friction velocity as the primary driving variable. The well-known four-parameter and six-parameter Iwan models and Valanis endochronic model fall into this category. It corrects the previous classification mistake that incorrectly placed Iwan-type models within velocity-dependent dynamic friction models.

1. Valanis model

The Valanis model (often called the intrinsic time friction model/endochronic model) was formally put forward by K. C. Valanis in 1971. This is a nonlinear theoretical model commonly used in the analysis of bolted connections and plastic structures [61,62,63]. It summarizes the microscopic and macroscopic slip behavior of a contact interface through a set of equations. By adjusting the parameters and optimizing with MATLAB ABQ2015, the characteristics of a system under vibration can be better identified. The model expression is

(17) $$f_{\upsilon }=\frac{E_{0}\overset{•}{u}\left[1+\mathrm{sgn}\left(\overset{•}{u}\right)\frac{\lambda }{E_{0}}\left(E_{t}u-F\right)\right]}{1+\kappa \mathrm{sgn}\left(\overset{•}{u}\right)\frac{\lambda }{E_{0}}\left(E_{t}u-F\right)}$$

(18) $$\lambda =\frac{E_{0}}{\alpha_{0}\left(1-\kappa \frac{E_{t}}{E_{0}}\right)}$$

**Figure 9:**
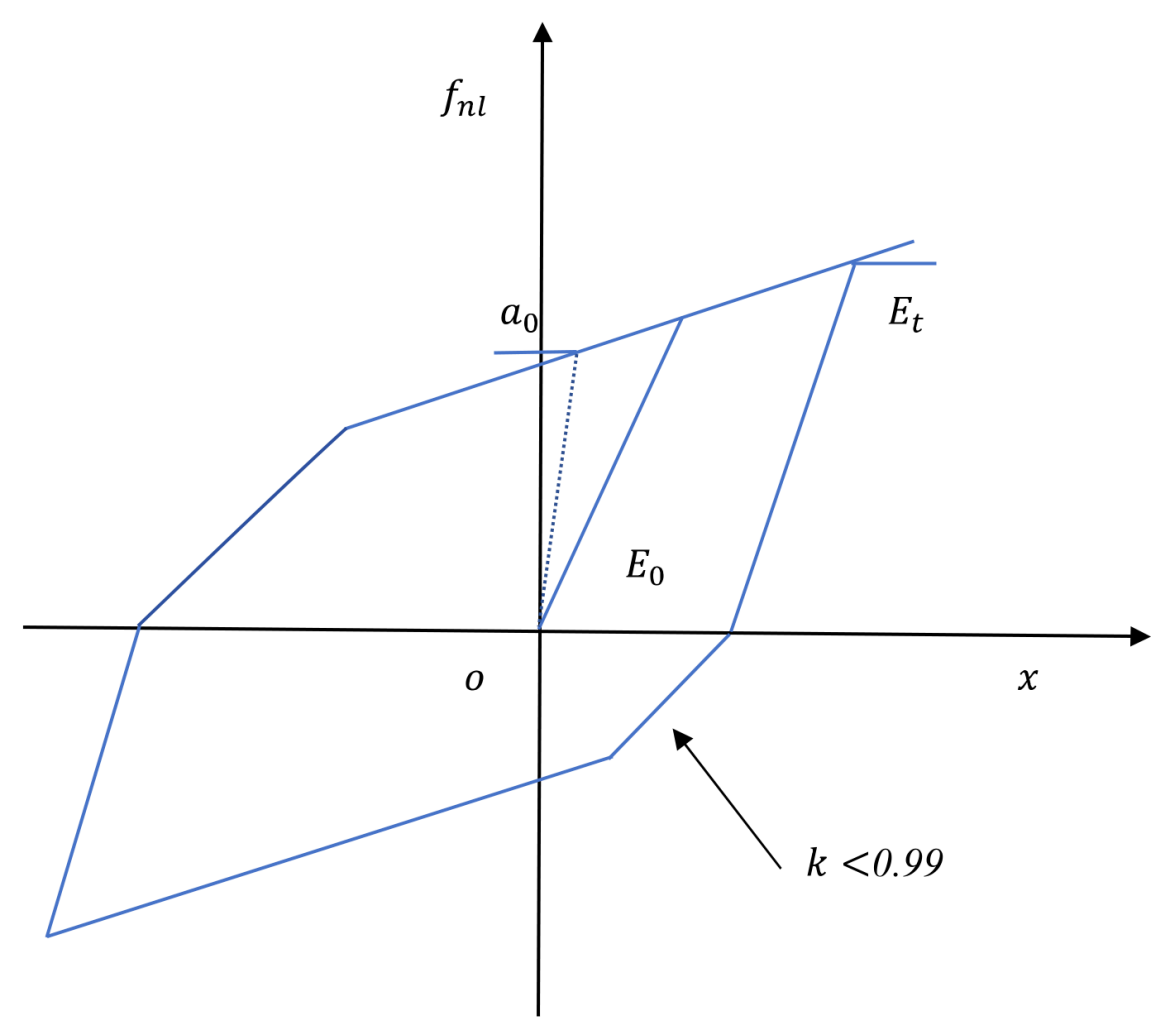
Working principle of Valanis model.

In the formula, $f_{\upsilon },u$ are the restoring force and displacement; $E_{0},E_{t}$ are the parameters of the material; κ is a dimensionless parameter; and $\alpha_{0}$ is the yield point parameter. By setting the different parameters according to the requirements, the nonlinear behavior of a structure under external forces can be observed. After parameter processing and optimization through MATLAB, the accurate identification value under the excitation state can be obtained [64]. Its working principle is shown in Figure 9.

2. Iwan model and its application in bolt connections

**Figure 10:**
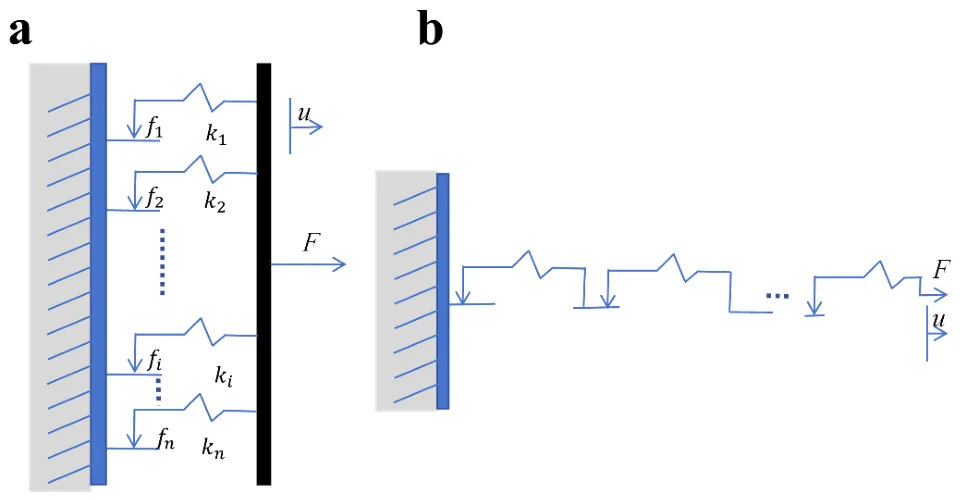
Iwan model: (**a**) Parallel–serial, (**b**) serial–Serial.

The Iwan model is based on a “bilinear hysteretic” idea, assuming that a friction system consists of many simple elastic–plastic elements [65,66]. Its structure is simple, and the theoretical results are in good agreement with the experimental data. The Iwan model mainly has two structures, series and parallel, as shown in Figure 10. The expression of the model is

(19) $$F=\int_{0}^{k_{t}x}f^{*}\varphi \left(f^{*}\right)df^{*}+k_{t}x\int_{k_{t}x}^{\infty }\varphi \left(f^{*}\right)df^{*}$$

where $k_{t}$ is the element stiffness, *x* is the tangential displacement, and $f^{*}$ is the element yield force.

#### 3.1.4. Reduced-Order Joint Equivalent Models

Reduced-order joint equivalent models do not refine the internal physical process of contact interface. Instead, they adopt lumped macroscopic stiffness–damping elements or low-order mathematical expressions to equivalently characterize joint nonlinearity, aiming at improving the computational efficiency for large-system simulations. They sacrifice local interface details for global calculation performance.

### 3.2. Detailed Review of Each Category of Models Under Above Unified Framework

Based on the above classification criteria, this subsection conducts a detailed literature review for each model category, summarizing their development progress, parameter physical meaning, advantages and inherent limitations when applied to bolted flange joints.

In general, the static friction model is relatively simple and has few parameters, but it cannot accurately describe the friction behavior when an object is moving, and it cannot reflect the hysteresis effect of friction. Therefore, researchers began to pay more attention to the dynamic friction model.

Static friction models have simple forms and low calculation costs, but they cannot characterize the preslip hysteresis or amplitude-dependent nonlinearity. The Dahl model realizes continuous hysteresis description without static friction consideration. The LuGre model balances computational efficiency and friction characterization capacity, yet suffers from numerical stiffness during velocity reversal. The four-parameter and six-parameter Iwan models are the most widely used in bolted joint dynamics, but their model parameters are mathematical fitting coefficients without a direct correspondence with physical parameters, such as bolt preload and flange stiffness. The Valanis model can simulate asymmetric hysteresis loops, but its intrinsic time theory results in a heavy calculation burden. None of the above models can naturally couple the time-varying preload loss caused by gasket creep.

From a micro–macro coupling perspective, a gradual interface microslip will reduce the effective contact constraints and contact stiffness, which will macroscopically manifest as structural stiffness attenuation and modal frequency drop. A reciprocating relative slip generates hysteretic frictional energy dissipation, leading to rising damping ratios with a growing excitation amplitude. The whole evolution of contact stick–slip state determines the nonlinear variation laws of global stiffness, damping and frequency response, and this micro–macro transmission chain is the core physical mechanism of bolted joint nonlinear dynamics.

Among various hysteretic constitutive models, the Iwan model is widely adopted in bolted joint dynamics research, which is the reason for its prominent position in this review. Its core advantage lies in the natural capacity to describe preslip stick–slip behavior and amplitude-dependent stiffness/damping variation, which matches the typical nonlinear dynamic characteristics of flange connections under cyclic vibration. Furthermore, the Iwan model can be conveniently calibrated via standard hysteresis experiments and readily reduced into low-order forms for system-level simulation. Nevertheless, this model cannot characterize velocity-dependent friction effects and struggles to capture asymmetric hysteresis loops induced by gasket creep. It should be noted that the advantages of the Iwan model are confined to vibration-induced microslip problems; for research dominated by sliding friction and velocity effects, the LuGre and Dahl models possess greater applicability.

The Iwan model is a commonly used model to study friction and bolt connections. Its classical parallel–series structure has wide applicability [67,68,69], and can also better reflect the physical principles behind it. But there is a problem with this basic model; that is, it cannot describe the part of the stiffness (that is, “residual stiffness”) that remains after a system slides. For this reason, Song specifically improved the nonlinear characteristics of bolted structures and established a new Iwan model that can include the residual stiffness [69].

In addition, the traditional Iwan model usually assumes that the compression force (normal load) between two objects is constant, which limits its application. In order to study the influence caused by a change in the compaction force, Rajaei [70] proposed a “generalized Iwan model” to deal with the changing normal load, and the effectiveness of this model was verified by testing a beam with friction support.

In order to describe the relationship between the friction energy consumption and load more accurately, Segalman [71] proposed a four-parameter Iwan model. This model contains key parameters, such as the force and stiffness needed to trigger macro-sliding, and can predict the relationship curve between the force and displacement at different times, but it still has difficulty to giving the residual stiffness accurately. Later, Li [72] further developed this line of research and proposed a six-parameter model. This model can distinguish the starting point of microslip and macroslip in more detail, and describe the change in stiffness and the final residual stiffness during sliding, so it is more descriptive than the four-parameter model.

Another practical problem is that the contact area of bolted connection structure will actually change when it is subjected to a lateral force, but most Iwan models assume that this area remains unchanged. In view of this, Wang clearly divided Iwan models into two categories: dynamic Iwan models consider the change in the contact area and pressure, and static Iwan models assume that they remain unchanged [73,74,75]. For the dynamic model, he put forward the corresponding mathematical expression and solution method. For the static model, the key mechanical response curve was deduced, and the accuracy of the model was verified by experiments and simulations.

In recent years, researchers have explored the application of Iwan model. Liu Bing [76] put forward a method for establishing a parameterized Iwan model with classical ANSYS software ABQ2022; Zhu Mingyang [77] used the six-parameter Iwan model to model and identify the parameters of a bolted connection structure. Generally speaking, the Iwan model has good applicability and can describe the dynamic characteristics of bolted structures, but it also has some shortcomings: the meaning of some parameters is not clear enough, some assumptions in the model are too simplified, and some real physical details are ignored.

To quantitatively illustrate the practical application status of above-mentioned constitutive models in bolted flange degradation research, a statistical analysis is carried out based on the included literature dataset of this review. A quantitative analysis of the adoption frequency of mainstream constitutive models across the 94 screened publications on cyclic flange joint degradation shows that: 45 studies (47.9%) apply the Iwan model for interfacial stick–slip characterization, 17 studies (18.1%) utilize the LuGre velocity-dependent friction framework, and nine studies (9.6%) adopt the Valanis endochronic model. The rest of the literature relies on basic static friction models or simplified reduced-order lumped parameter equivalents.

The statistical discrepancy originates from the inherent model characteristics. The Iwan series model takes the partial microslip evolution as its core physical mechanism, and naturally matches the full-stick–partial microslip–macroslip degradation process of flange interfaces under cyclic transverse loads. It can output closed hysteresis loops with clear physical parameters and low computational cost; hence it has become the most widely adopted model for cyclic vibration-induced degradation research of bolted flange joints [78]. By contrast, the LuGre focuses on velocity-dependent friction behavior and is more suitable for variable-speed friction research; the Valanis endochronic model suffers from higher parameter identification difficulty, limiting its popularization in flange engineering degradation analysis.

To conduct a fair comparison, all models are evaluated under consistent criteria, covering preslip characterization, velocity effect, amplitude dependence, parameter interpretability, calculation cost and applicability to bolted connection problems, as listed in Table 2.

The Stribeck and LuGre focus on velocity-varying sliding friction, whereas the Iwan and Valanis prioritize cyclic hysteretic behavior induced by interface stick–slip. Such inherent differences explain why certain models perform well for sliding friction problems but are less suitable to describe bolt joint dynamic degradation. Most existing studies select models without standardized comparison criteria, easily resulting in inappropriate model adoption.

### 3.3. Interface Degradation Mechanism

**Figure 11:**
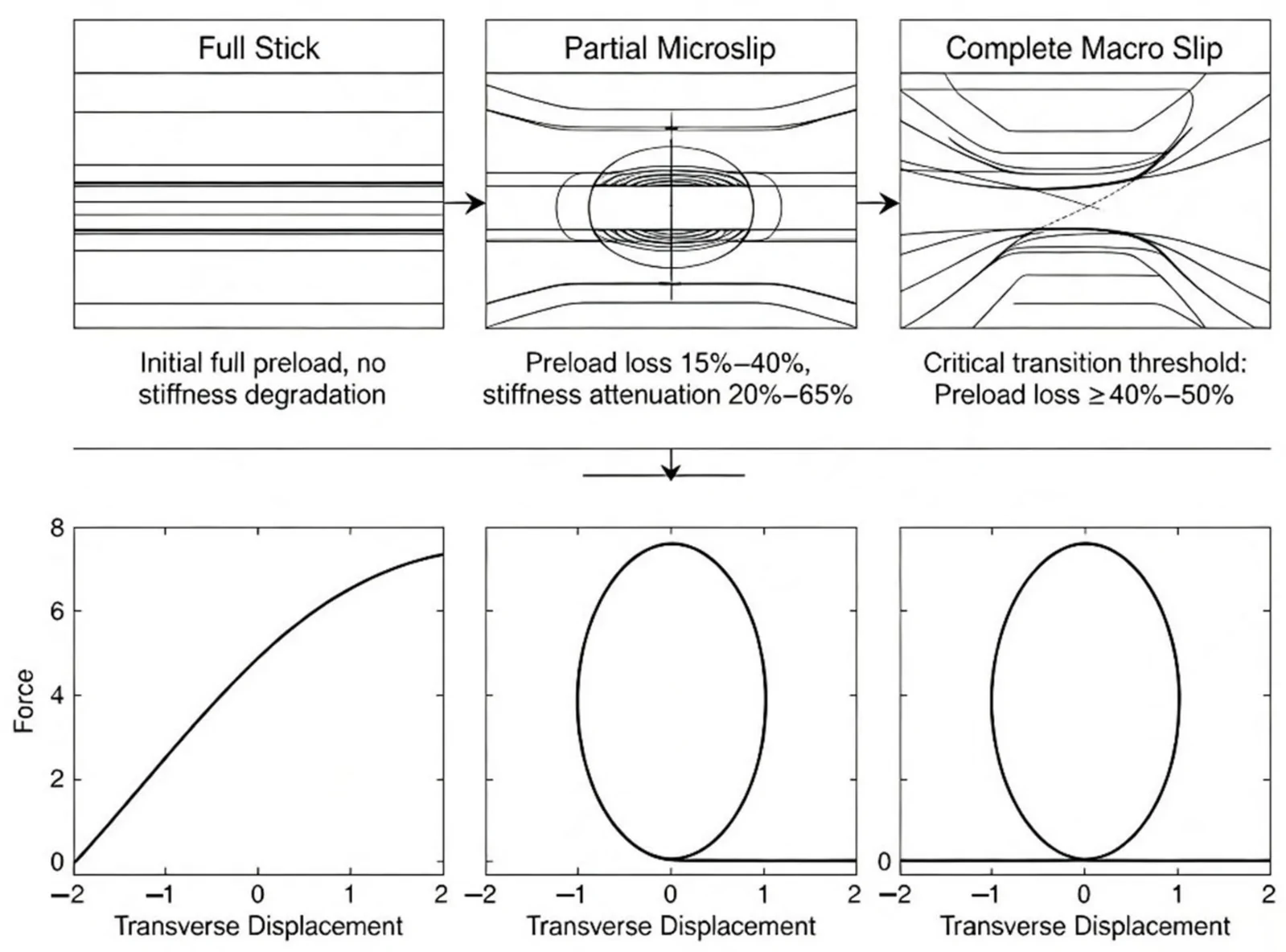
Schematic diagram of flange interfacial contact state evolution and corresponding force–displacement hysteresis curve variation under cyclic transverse loading. Three typical evolutionary stages (full stick, partial microslip, complete macroslip) are displayed, illustrating the micro-contact asperity state, critical preload loss threshold, and morphological changes in hysteresis loops during progressive slip transition.

At the micro–macro coupling level, the mechanical evolution of bolted flange interfaces under cyclic transverse loading can be divided into three successive stages: full stick, partial microslip, and complete macroslip. In the full-stick stage, the contact asperities over the flange interface remain stuck without relative tangential motion. The initial tangential stiffness maintains a high level, and nearly no energy dissipation occurs within the hysteresis loop, while slow preload loss is mainly induced by local asperity creep and thread friction wear. As cyclic loading accumulates and the bolt preload gradually decays, the contact periphery begins to experience localized slip while the central contact region remains adhered, entering the partial microslip stage. With the progressive loss of preload, the initial tangential stiffness degrades quantitatively: experimental observations report that a 15–25% preload loss typically leads to a 20–40% reduction in the initial tangential stiffness; when the preload attenuation reaches 30–40%, the initial stiffness may drop by 45–65% compared with the pristine assembled state [67]. A further preload reduction triggers the transition toward the complete macroslip stage, where large-scale relative tangential displacement occurs across the contact interface. According to existing experimental measurements for metallic flange–bolt connections, the critical preload loss threshold for the transition from partial microslip to macroslip generally falls within the range of 40–50% of the original assembly preload [65]. Throughout the three-stage evolution, the shape of force–displacement hysteresis loops changes correspondingly: narrow nearly linear loops for full stick; plump, rounded hysteresis branches for partial microslip; and low-stiffness large-opening loops for complete macroslip, as illustrated in Figure 11. This sequential contact state transition provides the physical basis for the time-varying updating of Iwan’s four parameters under preload relaxation.

### 3.4. Parameter Identification and Limitation of Theoretical Model

Parameter identification is a critical practical bottleneck restricting the engineering application of physics-based joint constitutive models. Even models with accurate interfacial stick–slip descriptions will produce unreliable degradation predictions if poorly calibrated due to discrepancies in objective function design, anti-noise performance and cross-condition generalizations capacity. Based on the unified classification framework established in Section 3.1, this subsection systematically compares three mainstream hysteresis and dynamic friction models: the Iwan microslip model, LuGre velocity-dependent friction model, and Valanis endochronic model. Four core evaluation dimensions are adopted: parameter identification difficulty, objective function construction rules, anti-noise robustness and cross-working-condition transferability, with the representative literature cited to support each comparative conclusion.

#### 3.4.1. Iwan Model

Parameter identification is generally implemented based on quasi-static force–displacement hysteresis loops obtained under cyclic transverse loading [79]. The standard identification workflow adopts least-squares fitting between the simulated and experimental hysteresis branches, taking the loop area, stiffness degradation, and slip displacement as joint objective functions. However, due to the ill-posed characteristics of multi-parameter collaborative optimization, multiple groups of parameter sets can fit noisy hysteresis data equivalently, resulting in non-unique identification solutions [80]. Furthermore, the identified Iwan parameters exhibit strong path dependence on the excitation amplitude and loading history. The model parameters calibrated under one specific load level cannot be directly transplanted to other amplitude conditions, which limits its cross-condition generalizations ability.

#### 3.4.2. LuGre Model

Time-domain dynamic response data are required for parameter calibration, and the objective function is usually constructed based on vibration response errors and friction force residuals in continuous time series [52]. Nevertheless, the LuGre model presents with prominent noise sensitivity during identification. High-frequency thermal noise and long-term zero drift easily interfere with the state variable iteration process, resulting in obvious parameter oscillation. In particular, numerical stiffness occurs frequently during velocity reversal stages under cyclic loading, which significantly reduces the robustness and stability of identification results [79]. Therefore, LuGre identification usually requires strict signal filtering and high-precision synchronous sampling conditions.

#### 3.4.3. Valanis Model

This model possesses relatively fewer intrinsic physical parameters and avoids the multi-solution problem of multi-parameter coupling optimization to a certain extent [61]. However, its highly nonlinear endochronic integral framework leads to a heavy iterative computational burden during calibration. In addition, Valanis model parameters lack explicit direct mapping with bolt preload and interfacial contact states. Once the flange preload attenuates or the contact pressure distribution changes, the previously identified parameters fail to maintain prediction accuracy, resulting in poor cross-condition transferability under variable preload degradation scenarios [81].

A comparative summary of identified bottlenecks for the three mainstream models are as follows. In general, each model exhibits distinct inherent limitations in parameter identification and engineering applicability. The Iwan model suffers from non-unique parametric solutions and load–amplitude path dependence, restricting its generalizations under variable cyclic excitation. The LuGre model is limited by weak anti-noise robustness and numerical instability during velocity reversal, demanding high-quality experimental signals. The Valanis model is restricted by high computational cost and poor adaptability to preload variation. These identified bottlenecks further explain why the time-varying degradation monitoring of bolted flange joints still lacks a unified, robust, and widely transferable constitutive identification framework, providing a clear motivation for improving time-varying model calibration strategies for multi-bolt flange structures.

To sum up, for the phenomenological models represented by the Iwan model, the assumption of their core density function is unreasonable, and many parameters are mathematical coefficients obtained by fitting experimental data, which are not directly related to the physical parameters that designers really care about, such as bolt preload, flange stiffness and material elastic modulus. This leads to an embarrassing situation: even if accurate parameter identification is completed, it is difficult for a design engineer to directly answer engineering questions, such as “how tight to screw” or “whether the flange stiffness design is reasonable” [77]. Most of the above models are essentially “calibration models”; that is, after calibration under a nominal load, they can make a good prediction of the response in the vicinity of the load, but their parameters lack extrapolation ability. Once the load range changes significantly or the loading conditions switch (such as changing from monotonous loading to cyclic loading), the previously identified parameters are no longer effective. Brake et al. clearly pointed out in their review article of the 2023 International Symposium on Mechanics of Connected Structures that, at present, the effective stiffness and damping of bolted joints cannot be accurately predicted with the best simulation tools, and the reliable prediction of nonlinear dynamics and wear characteristics is still far away from engineering designers [8].

In addition to the above common problems, different models also have their own unique shortcomings. The classical parallel–series structure of Iwan model cannot describe the residual stiffness of a system after sliding, and most models assume that the contact area is constant and the normal load is constant, which is different from the actual working conditions [76]. The LuGre model has numerical rigidity when the speed is reversed, and its accuracy is limited by the integrity of test data and the convergence of identification algorithm. Although the parameters of generalized Valanis model are more refined, the calculation amount required for intrinsic time theory is too large, and the mechanism’s clarity is still not as good as that of the model based on rough surface contact theory.

Parameter identification in friction and hysteresis models faces several inherent obstacles. First, there is parameter non-uniqueness: different parameter sets can generate nearly consistent force–displacement hysteresis loops under specific excitation, which lead to multiple feasible solutions during fitting. Second, there is path dependence: calibrated parameters are highly sensitive to the loading history, excitation amplitude and loading sequence; the parameters identified under one vibration condition cannot be directly transplanted to other working conditions. Third, measurement noise and sensor drift deteriorate the robustness of identification results. In addition, inconsistent interfacial contact states between repeated specimens reduce the experimental repeatability, further enlarging parameter deviation. The current identification methods mostly fit single-group hysteresis data, and few algorithms simultaneously constrain the above four limitations for multi-bolt flange structures.

## 4. Coupling Degradation Mechanism of Multi-Flange-Bolted System

However, most of the above studies are based on a single-bolt or simplified connection structure, which are significantly different from multi-flange-bolted systems in real engineering. In an actual service environment, the flange connection is a typical “multi-bolt cooperative work” system. Because the flange ring itself is not absolutely rigid, it will deform under the action of internal pressure and external bending moment, resulting in an extremely uneven load distribution on each bolt. The relaxation and degradation of a bolt will change the load distribution of the whole system, cause coupling effects, and may accelerate the relaxation and degradation of other bolts. This complex group coupling interaction is often oversimplified in the existing design specifications, and is only covered by a hypothesis of “safety factor” and “uniform preload”, which obviously cannot reflect the real and dangerous physical process. In order to make up for the shortcomings of the above research, researchers have focused on the elastic interaction and preload distribution from the simplified flange model, and finally clarified the degradation effect of multi-bolt coupling, gradually improving the mechanism research on the coupling degradation of multi-flange-bolted systems.

### 4.1. Study on Simplified Flange Model

At the beginning of the 20th century, with the wide application of flange connections between pressure vessels and pipelines in engineering fields, researchers conducted preliminary research on the problem of flange interface degradation. However, the early research on flange surfaces mainly focused on engineering requirements, such as sealing failure and functional failure. Between 1891 and 1911, Bach, a German scholar, first proposed the method of simplifying a flange into a cantilever beam to calculate the bending stress, but it was gradually replaced because of its oversimplification [82]. In 1937, Waters divided a flange into three parts—the flange ring, cone neck and shell—for an interaction analysis. Although the deformation was not directly calculated, it was still the theoretical basis of multinational standards to indirectly ensure rigidity and achieve sealing by controlling the “elastic nominal stress”. On this basis, researchers began to shift perspective from the flange itself to the “deformation coordination” of the whole connection system. In 1982, the American Welding Research Council issued research bulletin No.264, which deeply discussed the deformation coordination and sealing analysis of bolted flange connection systems [36].

In view of the high requirements of flange connection structures for water tightness and air tightness, applying a reasonable pre-tightening force has become the key index to ensure the normal function of flange structures. Nangong’s Self-Army [83] used contact theory and the finite element method to derive the formula for calculating the optimal pre-tightening force when there is an air tightness requirement on the flange butt surface, and its research conclusion has a certain engineering application value. The expression of its pre-tightening force is

(20) $$F=\frac{F_{0}L_{2}+\Delta L_{2}EA}{L_{2}-\Delta L_{2}}$$

where $F_{0}$ is the initial pre-tightening force, $L_{2}$ is the original thickness of the connected piece, $\Delta L_{2}$ is the compression amount of the connected piece after the pre-tightening force is applied, EA is the stiffness of the screw, *E* is the elastic modulus of the screw material, and *A* is the elastic modulus of the screw material.

Subsequently, Hao Zhengquan and others [39] derived the deformation compatibility equation of flange connection at a high temperature through the experimental study of the compression resilience characteristics, relaxation characteristics and basic sealing characteristics of flexible graphite composite gasket at a high temperature. The equation is as follows:

(21) $$(D_{k}-D_{G})-K_{B}(F_{B2}-F_{B1})-2h_{g}(\theta_{2}-\theta_{1})-2K_{p}\cdot P-3X_{t}=0$$

In the formula, $D_{G}$ and $D_{k}$ represent the compression of the gasket under the conditions of operation and pre-tightening, $K_{B}$ represents the elastic coefficient of the bolt material, $F_{B1}$ represents the bolt pre-tightening load, $F_{B2}$ represents the bolt operating load, $h_{g}$ represents the bolt operating load, $\theta_{1}$ and $\theta_{2}$ represent the rotation angle of the flange during pre-tightening and operation, $K_{p}$ represents the elastic coefficient of the flange material considering the compression swelling effect, *P* represents the medium pressure, and the elongation of the bolt at a certain temperature is $X_{t}$.

Generally speaking, simplified flange models provided an important starting point and basic tool for the theoretical analysis of flange connections, but their assumptions of “uniform preload” and “independent bolt” were fundamentally different from the real physical process of multi-flange-bolted systems. It was this deviation that drove the follow-up research towards more detailed elastic interaction analyses and multi-bolt coupling degradation modeling.

There exist essential mechanical discrepancies between simplified single-bolt joints and engineering multi-bolt annular flanges. Single-bolt structures bear independent axial/shear loads without inter-bolt load interaction, and their contact constraints remain stable under external excitation. In contrast, multi-flange-bolted systems feature flexible ring deformation, obvious elastic interaction between adjacent bolts, uneven circumferential contact pressure, and cross-coupled preload loss. The microslip, stiffness attenuation and energy dissipation of each bolt trigger whole-system load redistribution, forming unique group-coupled nonlinear behaviors that single-bolt models fail to reflect.

### 4.2. Elastic Interaction and Pre-Tightening Force Distribution

In order to meet the needs of engineering applications, Nassar et al. explored the law of preload loss caused by the elastic interaction and creep relaxation of sealing gasket in bolted flange connections under different tightening methods, sealing gasket types and flange structural parameters. Based on the beam theory on elastic foundations, Koves [84] equates the sealing gasket and flange ring to the beam structure on elastic foundations. At the same time, the influence of flange ring and taper neck curvature on the flange bending stiffness is considered. By solving the control differential equation displacement of elastic foundation beam, the evaluation of bolt spacing is realized. However, the circumferential bending and torsion effects of the flange are not considered in this method. In order to solve this problem, Abid et al. [85] built a linear spring model of flange-bolted connection, and analyzed the dispersion and relaxation characteristics of bolt pre-tightening force caused by elastic interaction and the performance change law of sealing gasket under different pre-tightening methods through simulations.

Nassar and Yang [41] deduced the elastic interaction flexibility of the sealing gasket of flange connection based on the displacement and deformation compatibility equation of bolt connection, assuming that the strain of the sealing gasket is symmetrical and the flange plane is not distorted, and then determined the residual pre-tightening force, and explored the influence of the tightening sequence, elastic modulus of the sealing gasket, gasket thickness and clamping length on the load, but their methods ignored the deformation effect of the flange plane. Okorn [86] further analyzed and calculated the elastic interaction effect of flange, bolt and sealing gasket under bending, torsion and axial compression on the basis of Koves, and gave a detailed solution method for a differential equation of circular beam deformation on an elastic foundation. This method has high accuracy, but the solution process for the differential equation is complicated. In the following research, the team deduced the stress distribution function of bolt joints, determined the stress–strain relationship through a Fourier series and boundary conditions, and then got the interaction stiffness, but the calculation method was complicated and inconvenient for engineering practice. Zhu et al. [26] used a three-dimensional finite element model to study the elastic interaction effect of bolted flange connections, and considered the nonlinear elastic behavior of gaskets in the numerical simulation, and verified the correctness of the pre-tightening force formula of elastic interaction bolts and the effectiveness of the simulation method through the neck-welded flange test of NPS4Class900. Li Ling et al. [44] verified the rationality of the pre-tightening force formula of elastic interaction under different pre-tightening sequences and load steps through a finite element analysis, and explained the variation law of pre-tightening force in detail, but failed to derive an analytical solution that could be directly applied to engineering practice. Duan Hongyan et al. [36] established a simplified stress model of elastic interaction, and based on the coordination relationship between the stress balance and deformation, the formula for calculating the residual preload of bolted flange joints with stiffness as the core parameter was derived. However, the above elastic interaction model only takes the bolt layout into account, ignoring the creep and friction governing factors that dominate long-term coupled degradation evolution.

### 4.3. Multi-Bolt Coupling Degradation Effect

From the above, we can see that both the classical Waters method and the more detailed deformation compatibility equation are based on the core assumptions of “uniform preload” and “bolt independence”. However, when this theoretical framework faces the multi-flange-bolted system in real engineering, its inherent defects and theoretical limitations begin to be exposed. In order to provide a theoretical basis for the development of multi-bolt coupling degradation model, it is necessary to systematically clarify the physical essence and hierarchical embodiment of these limitations. The fundamental limitation of the single-bolt model lies in its implicit premise that the flange ring remains absolutely rigid under stress, so the preload of each bolt can be regarded as evenly distributed and not affecting each other. However, a great amount of engineering practice and research results have shown that this assumption is fundamentally different from the real physical process of flange connection. Because a flange ring itself is not an absolute rigid body, under the action of internal pressure, external bending moment and uneven load in the assembly process, significant circumferential bending and torsion deformation will occur, resulting in a significant uneven distribution of the bearing state of each bolt.

Abid et al. [85] revealed the dispersion and relaxation characteristics of bolt pre-tightening force caused by elastic interaction through a finite element simulation, and the performance change law of sealing gaskets under different pre-tightening methods. Li et al. [87] used a finite element numerical simulation method to study the elastic interaction law of three specifications of PN40 neck-welded flanges under cross and sequential loading, and systematically characterized the elastic interaction by establishing the elastic interaction coefficient matrix. When an individual bolt degrades prematurely due to insufficient pre-tightening or relaxation, its bearing capacity will be reduced, which will break the original load balance and force the system to redistribute the load originally borne by that bolt to the adjacent bolts. This load redistribution effect is further amplified under flexible deformation of the flange ring. Zheng Xiaoya et al. [88] analyzed the influence of different tightening techniques on the magnitude and dispersion of bolt pre-tightening force by establishing fine finite element models of flange connection structures with different threaded angle-rising bolts, and found that the load distribution of bolts after pre-tightening is determined by the distribution of the times of elastic interaction on each bolt, and the more times of elastic interaction on each bolt, the smaller the pre-tightening force.

Chen [89] systematically studied the elastic–static characteristics of bolted flange connections by combining a finite element analysis with experimental tests, and found that the axial stiffness showed remarkable bilinear characteristics, and the flange showed axial–bending coupling deformation under a bending load. The essence of this coupling deformation stems from the fundamental difference in the interface contact state between the connecting surfaces in tension and compression. The study further pointed out that flange-bolted axial stiffness and bending stiffness increase with an increase in bolt pre-tension, but the increase range and law are different. This further shows that in a multi-system, “cooperation” in the geometric sense not only means load distribution, but also means the nonlinear coupling deformation and stiffness evolution, which are physical processes that cannot be described by the single-bolt model.

It can be seen from the above systematic review of the theoretical models of connection structures that even in idealized studies of single-bolt connection, the parameter identification of mainstream theoretical frameworks, such as the Iwan model, LuGre model and Valanis model, all face common problems, such as vague physical meaning, dependence of calibration results on loading path and non-uniqueness of parameters. When these models are directly transplanted to multi-flange-bolted systems, their limitations are further amplified. On the one hand, the flexible deformation of flange in multi-bolt system introduces additional geometric and contact nonlinearity, which makes the interface friction energy consumption, stiffness degradation and pre-tension relaxation form multiple couplings, so it is difficult for any single model to cover all the physical processes at the same time. On the other hand, a multi-bolt system significantly increases the dimension of parameters to be identified compared with a single-bolt system, which makes the model parameters based on single-bolt data fitting no longer applicable to multi-bolt scenarios, and it is urgent to develop a modeling strategy with cross-scale and multi-parameter joint identification ability.

Although the time-varying Iwan model originally originated from single-bolt lap joint research, practical complex equipment structures widely adopt multi-bolt annular flange connections. Essential mechanical differences exist between single-bolt lap joints and multi-bolt annular flanges, as summarized in Table 3. For multi-bolt annular flanges under bending loads, finite element simulation results for neck-welded flanges demonstrate obvious circumferential unevenness of contact pressure. The contact pressure near bolt-hole regions maintains a relatively high magnitude, while pressure drops are evident at the mid-span position between two adjacent bolts; quantitatively, the contact pressure at the mid-span can decrease by 35–55% compared with the bolt-hole vicinity [89]. Different from single-bolt joints, multi-bolt flange systems exhibit distinctive degradation-chain-reaction behavior driven by inter-bolt elastic interaction. The degradation chain can be described as: local preload relaxation of individual bolt → load redistribution among adjacent bolts → accelerated global stiffness degradation of the whole flange joint [90]. Once one bolt suffers preload loss, the surrounding bolts will assume the redistributed additional load, which will further accelerate their own preload attenuation. Such a coupling propagation effect cannot be observed in single-bolt lap joint specimens, which indicates that directly transplanting single-bolt-identified time-varying Iwan parameters to multi-bolt annular flanges will result in obvious prediction deviation.

Table 3 presents a comparison of mechanical- and degradation-related characteristics between a single-bolt lap joint and multi-bolt annular flange joint. The circumferential contact-pressure difference in neck-welded flanges under a bending load and multi-bolt-specific degradation-chain-reaction behaviors are included.

Based on the above research, it is clear that there are still some shortcomings in the existing research on the coupling degradation effect of multi-bolts: First, the research system for studying the physical evolution mechanism of nonlinear coupling degradation is not clear enough. Second, most research still relies on the linear theory of elastic interaction and the empirical safety margin to design and check multi-flange-bolted systems, lacking an effective theoretical means to systematically characterize the internal physical process of coupling degradation. Thirdly, there is still a lack of systematic research on the quantification and spatial distribution characteristics of coupling strength and the attenuation law of the overall mechanical properties of a structure in the process of multi-bolt group coupling. Fourth, in the surveyed literature, the existing experimental testing devices and standard specimens are still mainly single-bolt or few-bolt systems, which cannot truly reproduce the coupling degradation behavior of multi-flange-bolted system under a complex load history, which means the theoretical model’s verification lacks a reliable experimental basis.

The load redistribution and coupled degradation of multi-flange-bolted systems are jointly controlled by three categories of core governing factors. The geometric factors include bolt spacing, flange ring rigidity and gasket thickness, which determine the intensity of elastic interaction between bolts. The material factors cover the gasket creep property, interface friction coefficient and bolt elastic modulus, which dominate the speed of preload decay and interfacial microslip. The external load factors comprise the cyclic vibration amplitude, internal medium pressure and temperature cycling, which accelerate the chain-type degradation transfer among bolt groups. Most existing analytical models only consider partial factors and fail to fully couple their combined effects.

The current research on multi-bolt coupling degradation still remains at the stage of qualitative phenomenon description and finite element numerical observation. Based on the included studies, quantitative characterization indexes of coupling strength, coupling transfer path and degradation acceleration coefficients have not been formed. The coupling degradation mechanism has only been explained from the perspective of elastic load redistribution, without considering the nonlinear superposition effect of interface friction hysteresis and creep evolution. Few mechanism–data hybrid degradation characterization models targeting annular multi-bolt flange structures have been reported in the current public literature.

### 4.4. Machine Learning, Digital Twins, and Physics-Informed Modeling

Recently, data-driven hybrid modeling strategies, including machine learning, digital twins and physics-informed neural networks, have opened new avenues for multi-bolt flange degradation prediction. Traditional pure data-driven models require massive labeled experimental datasets and lack clear physical interpretability, while standalone constitutive models struggle to capture complex coupled degradation phenomena. Hybrid frameworks embedding mechanical governing equations into data-driven algorithms can effectively reduce data dependency and avoid physically unreasonable prediction outputs. Within the scope of the reviewed literature, most experimental validations for such hybrid approaches have been conducted on simplified single-bolt laboratory specimens; systematic implementations of full-scale multi-bolt annular flanges subjected to complex coupled loads are still limited. Further dedicated research is required to promote their practical engineering applications. Comprehensive quantitative comparisons of their generalization capacity under variable preload conditions remain an open topic for follow-up studies [91,92,93].

### 4.5. Matching and Comparative Synthesis of Test Facilities, Sensing Techniques and Constitutive Models

Clear coupling relationships exist among the test platforms, sensing hardware, measurable physical quantities, and constitutive model calibration requirements in flange joint degradation research. Single-bolt standard test benches (e.g., Junker vibration testers) are convenient for offline model calibration, yet they cannot reproduce the circumferential bending deformation and inter-bolt elastic coupling that are intrinsic to annular flanges. Various sensors, including FBG strain sensors, piezoelectric sensors, eddy current displacement sensors, and acceleration sensors, each possess exclusive merits; nevertheless, they suffer from inherent drawbacks, such as long-term signal drift, multi-channel synchronization error, and physical installation constraints [36,38,39]. These practical imperfections can distort the measured hysteresis experimental data and further induce non-unique solutions in model-parameter-identification workflows.

Different categories of constitutive models impose distinct matching requirements for measured experimental signals. Hysteresis stick–slip models (Iwan-type and Valanis endochronic models) rely on complete force–displacement hysteresis curves obtained from synchronous measurements of tangential force and interfacial relative displacement. In contrast, velocity-dependent dynamic friction models (LuGre and Dahl models) prioritize high-quality time-domain velocity–friction time-series signals for reliable calibration.

Readers may refer to Table 1 for the performance summaries and inherent limitations of typical bolt joint testing equipment, and consult Table 2 for the comprehensive evaluation indicators of mainstream friction and hysteresis constitutive models to complete the cross-reference matching analysis. A dedicated independent synthesis table covering all matching relations is not provided herein to avoid deviating from this review’s core theme of interfacial constitutive degradation modeling.

## 5. Conclusions

### 5.1. Main Systematic Review Findings

This review summarizes the state-of-the-art research on the degradation mechanism and constitutive characterization of bolted flange joints under cyclic loading. The main findings are grouped into four modules concerning experiments, constitutive models, multi-bolt interaction and sensing techniques.

(1) Experimental test module: The typical test platforms for bolt joint degradation cover single-bolt lap joint benches, Junker-type transverse vibration testers and multi-bolt flange test rigs. Each facility has clear boundaries regarding applicable loading modes, measurement defects and engineering adaptability. Under cyclic combined loads, bolted flanges experience sequential full-stick, partial microslip and macroslip evolution. Preload relaxation (without nut rotation) and rotational self-loosening are two distinct degradation mechanisms, where embedding, creep and elastic interaction contribute to time-varying preload loss. Sealing leakage is regarded as a secondary engineering failure derived from excessive mechanical degradation rather than an intrinsic core degradation mode.

(2) Constitutive model module: A unified classification framework for joint interface constitutive models is established, including static friction models, velocity-dependent dynamic friction models, hysteresis stick–slip models and reduced-order equivalent models. The conceptual difference between the Dahl model (without Stribeck term) and LuGre model is clarified. Among hysteresis models, the four-parameter Iwan model shows outstanding capability for reproducing stick–slip hysteresis, but suffers from non-unique parameter solutions and excitation amplitude path dependence during identification. The LuGre is sensitive to sensor noise and exhibits numerical stiffness during velocity reversal; the Valanis endochronic model has a heavy computational burden and poor transferability under a variable preload. The existing model comparison indicates that no single model can simultaneously satisfy high physical interpretability, anti-noise robustness and cross-condition generalizations for multi-bolt flange degradation prediction.

(3) Multi-bolt flange interaction module: Distinct from single-bolt lap specimens, multi-bolt annular flanges are dominated by inter-bolt elastic interaction. A non-uniform circumferential contact pressure emerges under bending loads. Local bolt relaxation triggers load redistribution among adjacent bolts and further accelerates overall stiffness degradation, forming a chain-reaction degradation effect. Geometric, material and loading factors jointly govern the load-redistribution behavior of multi-bolt systems.

(4) Sensing and measurement module: Representative sensing techniques, including FBG, piezoelectric, eddy current, ultrasonic and acceleration sensors, have their respective advantages, while long-term drift, channel synchronization error and installation constraints constitute the major bottlenecks for multi-bolt flange synchronous degradation monitoring. Measurement noise and signal drift will distort experimental hysteresis data and further induce non-uniqueness in constitutive model parameter identification.

Taken together, the above-summarized findings are all derived from the 94 systematically screened publications included in this review. Although considerable progress has been achieved in flange–bolt–joint degradation research, multiple unresolved challenges still persist across mechanism investigation, constitutive modeling, measurement identification and engineering verification domains, which deserve further exploration in future work.

### 5.2. Future Research Outlook

This section outlines the open research challenges and potential research priorities that have not been fully addressed within the existing reviewed literature. These prospective research directions are organized hierarchically from physical–mechanism exploration to practical engineering validation.

The hierarchical measurable research prospects are proposed from mechanism, modeling, measurement identification and engineering verification levels for time-variant degradation research of multi-bolt flange joints.

(1) Degradation mechanism level: Further quantitative tests are required to obtain critical preload loss thresholds for the transition from partial microslip to macroslip under multi-bolt coupling conditions. Quantify the chain-reaction propagation law induced by local bolt relaxation, and clarify the relative importance and interaction of geometric–material-loading influencing factors for multi-bolt load redistribution.

(2) Constitutive modeling level: Improve the time-variant Iwan-series modeling frameworks for multi-bolt annular flanges. Develop calibration strategies to mitigate the non-uniqueness of parameter identification under noisy experimental data. Establish explicit mapping relationships between real-time preload evolution and Iwan model parameter updating rules, and reduce the empirical dependence of existing time-varying models.

(3) Measurement and parameter identification level: Develop multi-channel synchronous sensing schemes adapted to time-variant degradation models. Optimize sensor layouts and signal-processing algorithms to suppress long-term drift and synchronization errors in multi-bolt flange monitoring. Construct joint identification frameworks that take sensing uncertainty into consideration.

(4) Further dedicated investigations into digital twin and physics-informed modeling are needed to facilitate practical health-monitoring applications for bolted flange structures.

Future research must also address the development of multi-physics integrated sensing systems for annular flanges. It is necessary to realize the decoupling of mixed monitoring signals induced by multi-bolt coupling degradation, so to achieve accurate online condition evaluations based on sensor measurement data. The theoretical framework summarized in this review can provide effective guidance for such sensor-oriented monitoring technologies. Subsequent research should further decouple the coupling relationships between interfacial microslip evolution and macroscopic stiffness, and damping and frequency response, to realize quantitative prediction of structural dynamic characteristics based on the real contact state. Meanwhile, it is urgent to establish theoretical frameworks that systematically distinguish the mechanical behaviors of single-bolt structures and multi-flange-bolted groups, and eliminate the prediction error caused by directly transferring single-bolt experimental conclusions onto multi-bolt engineering components. Advanced multi-objective identification algorithms should be developed to mitigate parameter non-uniqueness and path dependence, and improve the robustness of model calibration against measurement uncertainties. As such, efforts should be devoted to developing physics-informed digital twin platforms, which integrate friction hysteresis constitutive models and sensor monitoring data to achieve the accurate long-term degradation prediction of multi-bolt flange structures. Finally, research should quantitatively decouple the respective contributions of geometric, material and load governing factors to realize accurate prediction of multi-bolt group coupled degradation.

## Figures and Tables

**Table 1:** Performance comparison of typical bolted joint test setups.

Test Equipment	Measurable Parameters	Core Measurement Accuracy	Main Limitations	Applicable Research Scenarios
Junker transverse vibration tester	Bolt preload; lateral reciprocating displacement	Preload: ±1.0% [27]	Only single bolt; no bending/inner pressure loading	Single-bolt transverse vibration loosening test
Schatz-Analyse multi-functional system	Tightening torque; thread friction; axial preload	Torque: ±0.5%	Static loading only; unable to simulate long-term vibration creep	Static torque-preload calibration; friction coefficient measurement
Universal testing machine	Static stiffness; friction hysteresis curve	High-grade force measurement accuracy (0.5 class)	Lacks dynamic cyclic vibration excitation	Quasi-static interfacial friction and stiffness test
Multi-mode loosening test device	Axial/torsional/shear alternating load; residual preload	Preload: ±2.5% [26]	Slightly lower precision than imported equipment	Multi-load coupling loosening of small flange specimens
Fretting wear test apparatus	Tangential friction force; microslip distance	Displacement resolution: Nanometer-level [17]	Small stroke, cannot reproduce large-scale bolt relaxation	Interfacial fretting friction characteristic test

All accuracy and resolution data in this table correspond to static-calibration laboratory reference conditions (ambient temperature 25 ± 2 °C, relative humidity 50 ± 5%). These performance indexes are only valid within the rated measurement range; the dynamic measurement accuracy may degrade under high-frequency (>10 Hz) or extreme-temperature operating conditions. Torque accuracy for the Schatz-Analyse system follows the ISO 16047 equipment specification.

**Table 2:** Comparative evaluation of typical friction and hysteresis models under unified criteria.

Model	Captures Preslip Hysteresis	Describes Velocity-Dependent Friction	Amplitude-Dependent Nonlinearity	Parameter Physical Meaning	Computational Burden	Suitability for Bolted Flange Dynamics
Stribeck	No	Yes	No [47,48,49]	High [47,48,49]	Low [47,48,49]	Steady-state sliding friction
Dahl	Yes	No	Limited [53,54]	Medium [53,54]	Low [53,54]	Simple hysteresis without velocity effect
LuGre	Yes	Yes	Limited [56]	Medium [56]	Moderate [56]	General dynamic friction simulation
Iwan	Yes	No	Excellent [59,60]	Medium (slip unit distribution parameters are empirical fittings, but initial stiffness/critical slip force have direct correspondence with flange preload and contact state) [65,66]	Moderate [65,66]	Cyclic microslip & joint nonlinear dynamics
Valanis	Yes	No	Excellent [62,63]	Low [62,63]	High [62,63]	Asymmetric hysteresis loop prediction

Unified definition of rating standard: Excellent = widely verified by joint test, clear physical mapping; Medium = partial working condition applicability; Low = strong empirical fitting, weak direct physical correspondence. All evaluation conclusions are supported by the cited literature.

**Table 3:** Comparative characteristics of single-bolt lap joint and multi-bolt annular flange joint.

Comparison Item	Single-Bolt Lap Joint	Multi-Bolt Annular Flange Joint
Load transfer path	Single concentrated load transfer; relatively simple force chain [88]	Multi-path circumferential load transfer; coupling of bending–tensile–shear loads [89]
Flange circumferential deformation	No circumferential ring-type deformation	Obvious circumferential bending deformation under external bending moment [89]
Contact pressure uniformity	Contact pressure concentrates around bolt hole; no circumferential span-wise variation	Circumferentially non-uniform; mid-span pressure reduces by 35–55% relative to bolt-hole zone [89]
Inter-bolt elastic interaction	Non-existent	Strong elastic interaction among adjacent bolts; preload variation in one bolt alters others’ load status [89]
Degradation propagation characteristic	Degradation is isolated; no chain-spread effect	Degradation chain reaction: local bolt relaxation → adjacent bolt load redistribution → accelerated global stiffness degradation [90]

## Data Availability

No new data were created or analyzed in this study. Data sharing is not applicable to this article.
